# A Dynamic Model of Interactions of Ca^2+^, Calmodulin, and Catalytic Subunits of Ca^2+^/Calmodulin-Dependent Protein Kinase II

**DOI:** 10.1371/journal.pcbi.1000675

**Published:** 2010-02-12

**Authors:** Shirley Pepke, Tamara Kinzer-Ursem, Stefan Mihalas, Mary B. Kennedy

**Affiliations:** 1 Center for Advanced Computing Research, California Institute of Technology, Pasadena, California, United States of America; 2 Division of Biology, California Institute of Technology, Pasadena, California, United States of America; UT Southwestern Medical Center, United States of America

## Abstract

During the acquisition of memories, influx of Ca^2+^ into the postsynaptic spine through the pores of activated N-methyl-d-aspartate-type glutamate receptors triggers processes that change the strength of excitatory synapses. The pattern of Ca^2+^ influx during the first few seconds of activity is interpreted within the Ca^2+^-dependent signaling network such that synaptic strength is eventually either potentiated or depressed. Many of the critical signaling enzymes that control synaptic plasticity, including Ca^2+^/calmodulin-dependent protein kinase II (CaMKII), are regulated by calmodulin, a small protein that can bind up to 4 Ca^2+^ ions. As a first step toward clarifying how the Ca^2+^-signaling network decides between potentiation or depression, we have created a kinetic model of the interactions of Ca^2+^, calmodulin, and CaMKII that represents our best understanding of the dynamics of these interactions under conditions that resemble those in a postsynaptic spine. We constrained parameters of the model from data in the literature, or from our own measurements, and then predicted time courses of activation and autophosphorylation of CaMKII under a variety of conditions. Simulations showed that species of calmodulin with fewer than four bound Ca^2+^ play a significant role in activation of CaMKII in the physiological regime, supporting the notion that processing of Ca^2+^ signals in a spine involves competition among target enzymes for binding to unsaturated species of CaM in an environment in which the concentration of Ca^2+^ is fluctuating rapidly. Indeed, we showed that dependence of activation on the frequency of Ca^2+^ transients arises from the kinetics of interaction of fluctuating Ca^2+^ with calmodulin/CaMKII complexes. We used parameter sensitivity analysis to identify which parameters will be most beneficial to measure more carefully to improve the accuracy of predictions. This model provides a quantitative base from which to build more complex dynamic models of postsynaptic signal transduction during learning.

## Author Summary

Networks of neurons in the brain are connected together by specialized signaling devices called synapses. One way an active neuron relays its activity to other neurons is by releasing small amounts of chemical transmitters from its presynaptic terminals which induce electrical activity in postsynaptic neurons connected to it. Memories are formed when synapses in the network encoding the memory change their strength in order to stabilize the network. The decision whether or not a synapse becomes potentiated is controlled by delicate variations in the amount of Ca^2+^ ions that flow across the membrane at the postsynaptic site, and by the pattern of influx over time. The mechanisms of activation of regulatory enzymes that decode this Ca^2+^ signal have been extensively studied under laboratory conditions which are different from the conditions encountered inside a neuron. Therefore, we created a dynamic model of activation of one enzyme that is critical for learning by Ca^2+^. The model allows us to simulate activation of the enzyme within a biochemical milieu similar to what it will encounter at the postsynaptic site. It predicts unexpected behaviors of the enzyme *in vivo* and provides a framework for quantitative exploration of complex mechanisms of synaptic plasticity.

## Introduction

Calcium (Ca^2+^) is a critical second messenger in the brain. For example, it has long been recognized that Ca^2+^ influx through N-methyl-D-aspartate (NMDA) receptors initiates changes at synapses that enable us to form memories and to learn. Transient influx of Ca^2+^ through NMDA receptors triggers activation of complex protein signaling networks that regulate changes in synaptic efficacy including long-term potentiation (LTP) and long-term depression (LDP) [1],[2]. Calmodulin (CaM), a small protein (18 kDal) with four Ca^2+^ binding sites, is a molecular detector of influxes of Ca^2+^ across the synaptic membrane. It is ubiquitous in all cells including neurons [3]–[5], and it regulates proteins in postsynaptic spines of excitatory neurons [6]. When Ca^2+^ enters the spine, it binds to CaM and to other Ca^2+^-binding proteins. As Ca^2+^ binds to CaM, the Ca^2+^/CaM complex can then bind to and regulate its enzyme targets, many of which are immobilized in the “postsynaptic density” (PSD), a scaffold for signaling molecules attached to the postsynaptic membrane [7]. The relative rates of binding and affinities of the target enzymes for Ca^2+^/CaM are believed to determine their level of activity in a sensitive and selective fashion. Among the prominent CaM targets in the spine is Ca^2+^/CaM-dependent protein kinase II (CaMKII) [8], which plays a central role in initiating persistent synaptic changes [9]. It is required for normal LTP; transgenic mice lacking the major neuronal subtype of CaMKII show defective LTP and are deficient in spatial learning and memory [10],[11]. Thus, understanding the kinetics of interactions of Ca^2+^, CaM, and CaMKII can provide important insight into the initiation of mechanisms of synaptic plasticity.

**Figure 1:**
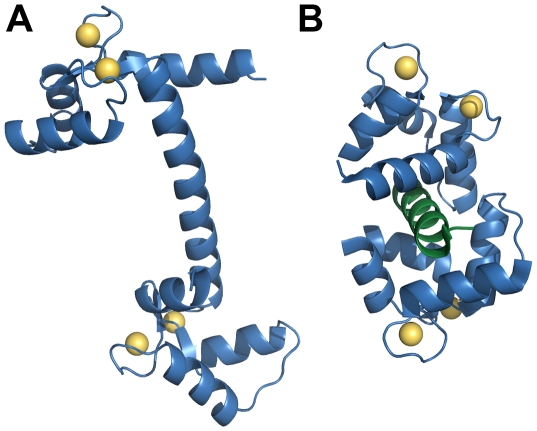
Structure of Calmodulin (CaM). (A) A 1.7 angstrom ribbon structure of free CaM (blue) with four bound Ca^2+^ ions (yellow). PDB 1CLL [56] (B) A 2.4 angstrom ribbon structure of CaM bound to a peptide (green) corresponding to the CaM binding domain of CaMKII. (Residues 74–83 of the CaM linker region are not resolved in this structure.) PDB 1CLL [15].

The structure and Ca^2+^ binding properties of CaM have been extensively characterized [12]. It comprises two pairs of Ca^2+^-binding EF-hand domains located at the N-and C-termini, respectively, separated by a flexible linker region (Figure 1, [13]–[15]). The pairs of EF-hands at the N and C termini have substantially different Ca^2+^ binding kinetics; however, both pairs bind Ca^2+^ ions cooperatively [16],[17]. The interactions of Ca^2+^-bound CaM with its targets are kinetically complex. CaM's affinity for many of its target proteins is increased upon Ca^2+^ binding and it's affinity for Ca^2+^ is enhanced upon binding of its target proteins [18]. Dissociation of the N-terminal bound Ca^2+^ ions from CaM often precedes dissociation of CaM from its target peptides [19]. When this is the case, the dissociation rate of the peptide from the Ca^2+^-bound C-terminal domain of CaM (CaM-2C) strongly influences the overall dissociation rate of the peptide from CaM. The kinetics of Ca^2+^ binding to CaM are likely to be particularly important in determining the outcome of Ca^2+^ signaling in neuronal spines because Ca^2+^ triggers biochemical events in the spine during a period when the Ca^2+^ concentration is fluctuating rapidly [20]. Furthermore, in conditions of limiting Ca^2+^, including basal Ca^2+^ or during relatively low amplitude Ca^2+^ transients, very few of the free CaM molecules will have four bound Ca^2+^ ions. Yet physiological responses to small increases in Ca^2+^ are observed [21], suggesting that CaM with fewer than 4 bound Ca^2+^ ions participates in initiating these responses.

CaMKII is a large holoenzyme comprising 12 homologous catalytic subunits held together by association of their carboxyl terminal domains [8],[22]. Each of the subunits contains a single CaM binding domain. Binding of Ca^2+^/CaM to this domain releases inhibition of the active site and stimulates catalytic activity [23]. Soon thereafter, a specific site within the catalytic subunit is autophosphorylated; the autophosphorylation event stabilizes the active conformation resulting in Ca^2+^-independent catalytic activity [24]. Recently, we showed that CaM with two Ca^2+^ ions bound to its C-terminal sites, binds to CaMKII and activates autophosphorylation, though at a ten-fold lower catalytic rate than fully loaded CaM (CaM with 4 Ca^2+^ bound) [25]. Thus, a kinetic model that describes Ca^2+^ binding to each of the individual CaM termini, as well as binding of Ca^2+^ to the CaM/CaMKII complex is important for a complete description of the activation dynamics of CaMKII in spines. Furthermore, a model that accounts for the activity of CaM/CaMKII with less than 4 bound Ca^2+^, is necessary to understand the extent of activation of CaMKII at relatively low and/or fluctuating Ca^2+^ concentrations such as occur in the spine cytosol.

Here we present two kinetic models of activation of monomeric subunits of CaMKII which include binding of Ca^2+^ to free CaM and to CaM bound to individual (ie. monomeric) CaMKII subunits. The model of monomeric CaMKII (mCaMKII) allows us to examine the significance of the dynamics of Ca^2+^ and CaM binding independent of the effects of cooperativity of binding of CaM between subunits within the holoenzyme [26]. Both models include the different kinetics of Ca^2+^/CaM binding at the N and C termini, and the thermodynamic stabilization of Ca^2+^-binding when CaM is bound to a target protein [18]. The first model is a complete model of binding of Ca^2+^ to the two CaM termini, including 9 Ca^2+^/CaM states and their interactions with mCaMKII. It differs from a recently published allosteric model [27] in which the Ca^2+^ binding rates depend explicitly on whether CaM is in one of two abstracted ensemble conformational states, R or T. Most of the required kinetic rates in our model are well constrained by previous experimental studies; however, a few have not been measured directly. In these cases, we used the principle of microscopic reversibility and fitting of existing experimental data to derive reasonable ranges of values for the kinetic rates. The second model is a coarse-grained model that is motivated by experiments showing high cooperativity of binding between Ca^2+^ ions at each terminus [16]. Binding of the second Ca^2+^ to each terminus of CaM is assumed to be rapid; thus, binding of pairs of Ca^2+^ to each terminus is treated as a single event. The resulting model includes 4 Ca^2+^/CaM states and their interactions with mCaMKII.

We created computer simulations based on each of these two models and explored their behavior under commonly used experimental concentrations of Ca^2+^, CaM, and mCaMKII, and under conditions that are closer to those believed to exist in synaptic spines. We determined a range of initial conditions under which the results of the coarse grained, pair-binding model are indistinguishable from those of the complete model, and a range under which the two deviate significantly. We show that Ca^2+^/CaM species with fewer than four bound Ca^2+^ predominate under many conditions that are believed to prevail in spines, and can sometimes completely determine the level of autophosphorylation. We find that activation of mCaMKII is highest at a particular frequency of Ca^2+^ fluctuations. The frequency that gives highest activation depends on the ratio of the time interval between Ca^2+^ transients and the rates of Ca^2+^ binding to the N and C termini of CaM, as well as on the the width of the Ca^2+^ transients. Finally, we performed global variation and sensitivity analyses to determine which parameters most affect the levels of autophosphorylation at particular times and under various conditions. We use these analyses to help infer the kinetic pathways through which autophosphorylation of CaMKII is likely to occur and to identify parameters whose refinement by direct measurement will be most important for the accuracy of predictions from our models.

The models presented here are a first step in a larger project to build kinetic simulations of activation of the CaMKII holoenzyme in the context of physiologically realistic models of Ca^2+^ fluctuations in postsynaptic spines [25],[28]. In addition, the models provide a framework in which to study activation of other Ca^2+^/CaM dependent enzymes, including the CaM-dependent protein kinases (CaMKI, CaMKIV, CaMKK, myosin light chain kinase), phosphatases (calcineurin) and others (adenylate cyclase, neuronal nitric oxide synthase, etc). Detailed kinetic analysis of these interactions are critical for understanding the molecular mechanisms that underlie synaptic plasticity because the events that determine whether a synapse undergoes LTP or LTD are determined under non-equilibrium conditions, when the Ca^2+^ concentration is fluctuating. Such analyses may also be useful for understanding Ca^2+^/CaM signaling in other tissues such as cardiac myocytes and cells of the immune system.

## Methods

### Models

We constructed a detailed model (Model 1) and a coarse-grained model (Model 2), both of which describe the kinetics of reversible binding of Ca^2+^ ions to free CaM and to the resulting intermediate Ca^2+^/CaM complexes. The models also describe reversible binding of Ca^2+^ to the Ca^2+^/CaM complexes after they have bound to individual subunits of CaMKII (mCaMKII). Finally, they describe the kinetics of irreversible autophosphorylation of mCaMKII, which is triggered by binding of Ca^2+^/CaM.

#### Model 1

**Figure 2:**
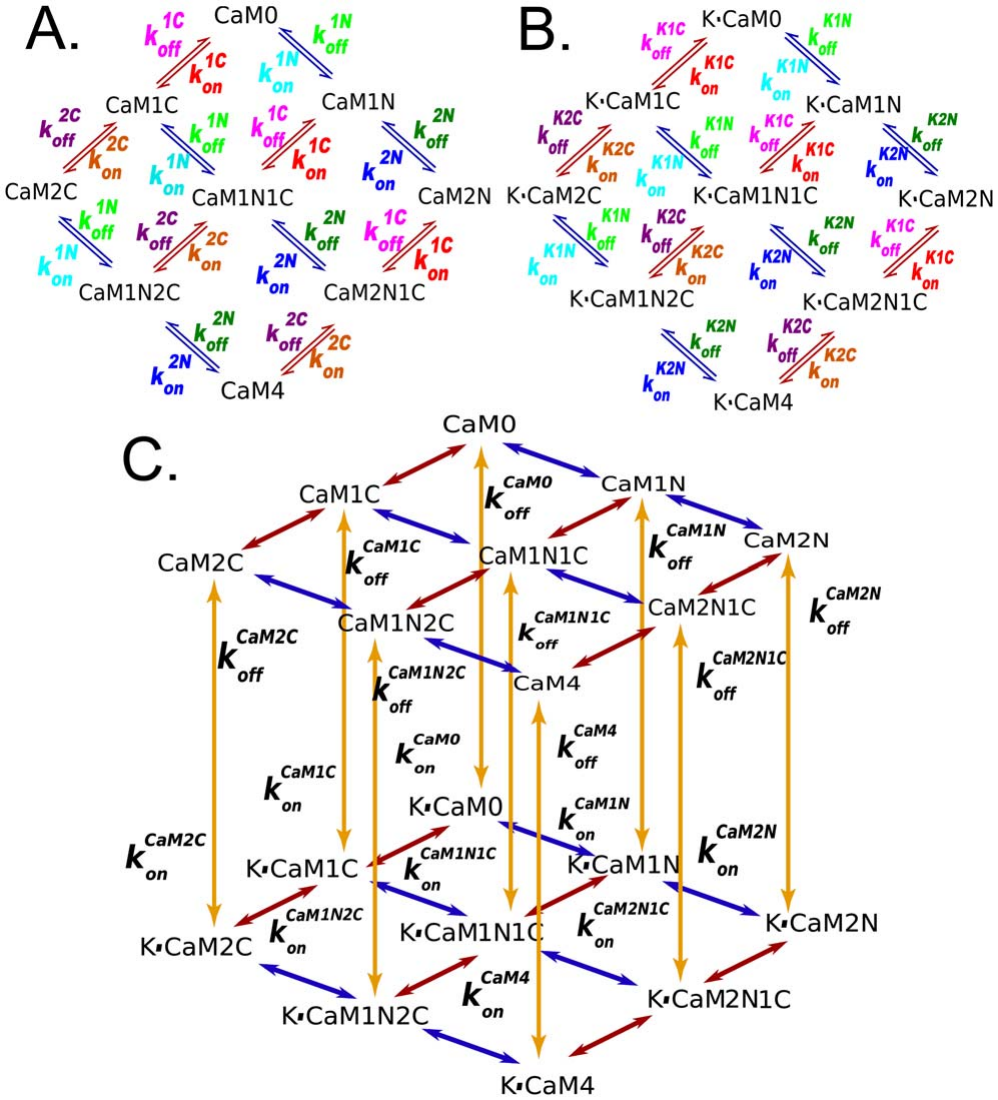
Model 1: binding among Ca^2+^, CaM, and mCaMKII. The top layer (A) represents binding of Ca^2+^ to CaM. Red arrows correspond to Ca^2+^ binding to the C-terminus, and blue arrows binding to the N-terminus. The bottom layer (B) represents Ca^2+^ binding to CaM while CaM is bound to CaMKII. The CaM species are denoted as CaM*n*N*c*C with *n*, *c* 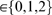, such that *n* is the number of Ca^2+^ bound to the amino (N) terminus and *c* is the number of Ca^2+^ bound to the carboxyl (C) terminus. The species of CaM bound to mCaMKII are denoted K•CaM*n*N*c*C. For convenience, we use CaM4 and K•CaM4 to denote species with *n = c = 2*, and CaM0 and K•CaM0 to denote those with *n = c = 0*. (C) The full model is represented as a cube, with yellow arrows indicating binding between CaM*n*N*c*C and mCaMKII.

Model 1 (Figure 2) is a “complete” nine-state model. It describes the binding of individual Ca^2+^ ions to the CaM termini, resulting in nine distinct Ca^2+^/CaM species or “states”, characterized by the number of Ca^2+^ ions bound at each terminus (Figure 2A and top layer, Figure 2C). The two sites that bind Ca^2+^ at a terminus are not distinguished from one another because previous investigators have shown that the sites at each terminus interact strongly upon Ca^2+^ binding; whereas the sites on opposite termini do not [16]. We use values from the literature, or our own measurements to constrain ranges of the parameters for the top layer of Model 1 and those for binding of CaM2C, CaM2N, and CaM4 between the layers in Figure 2C (See Text S1 and Figure S1 for complete derivations.).

We then use the thermodynamic principle of microscopic reversibility to constrain the equilibrium dissociation constants for the lower layer of the reaction model (Figure 2B), and for the remainder of the reactions between the layers, which represent interactions of CaM*n*N*c*C with mCaMKII (Figure 2C). Binding of CaM to CaMKII alters the affinity of CaM for Ca^2+^. Therefore, the 4 equilibrium constants (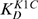,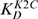,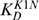,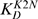) and 8 kinetic rates (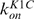,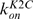,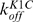,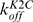,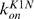 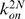,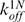,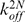) that specify binding of Ca^2+^ to K•CaM in Figure 2B (and Figure 2C, lower layer) are different from those that specify binding of Ca^2+^ to free CaM in Figure 2A (and Figure 2C, upper layer). The principle of microscopic reversibility states that the change in free energy around a reaction loop is zero and thus defines relationships among the equilibrium constants for Ca^2+^ in the upper and lower layers, and among those of the CaM*n*N*c*C species for CaMKII in the reactions between the layers (Figure 2C). We use the measured affinities of Ca^2+^ for free CaM and these relationships to constrain the affinities of CaM species for Ca^2+^ when they are bound to mCaMKII. In the same way, we use measurements of the affinities of CaMKII for CaM4, CaM2C, and CaM2N to constrain the affinities of CaMKII for CaM species with odd numbers of bound Ca^2+^. An example of one of these calculations is given in Text S1.

**Figure 3:**
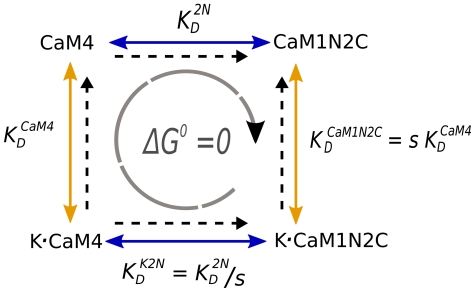
Energy loop diagram for derivation of cooperativity coefficients. The thermodynamic free energy around a reaction loop must sum to zero. This principle (microscopic reversibility) constrains the relationship between the equilibrium constants in the loop. We define cooperativity coefficients *s* (for the N-terminus of CaM) and *r* (for the C-terminus of CaM) to quantify the relationship between the affinity of Ca^2+^ for free CaM and of Ca^2+^ for CaM when bound to CaMKII. The principle of microscopic reversibility indicates that these coefficients also quantify the relationship between the affinity of CaMKII for CaM with three bound Ca^2+^ ions, and the affinity of CaMKII for CaM4, as shown in the figure for the N-terminal coefficient *s*.

To quantify the change in affinity of CaM for Ca^2+^ after CaM binds to mCaMKII, we define cooperativity coefficients *s* and *r*. The coefficient *s* represents the increase in affinity of the N lobe of CaM for Ca^2+^ when CaM binds to mCaMKII. Explicitly, *s* is the ratio of the dissociation constant of Ca^2+^ from CaM1N2C to the dissociation constant of Ca^2+^ from K•CaM1N2C, i.e., 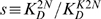 (Figure 3). The analagous coefficient *r* for the C lobe of CaM is defined as 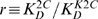. Again, using the principle of microscopic reversibility (Figure 3), we show that *s* and *r* also represent the proportional decrease in affinity of CaM for mCaMKII when CaM4 loses a Ca^2+^ to become CaM1N2C or CaM2N1C; thus, 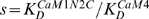, and 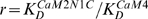.

**Figure 4:**
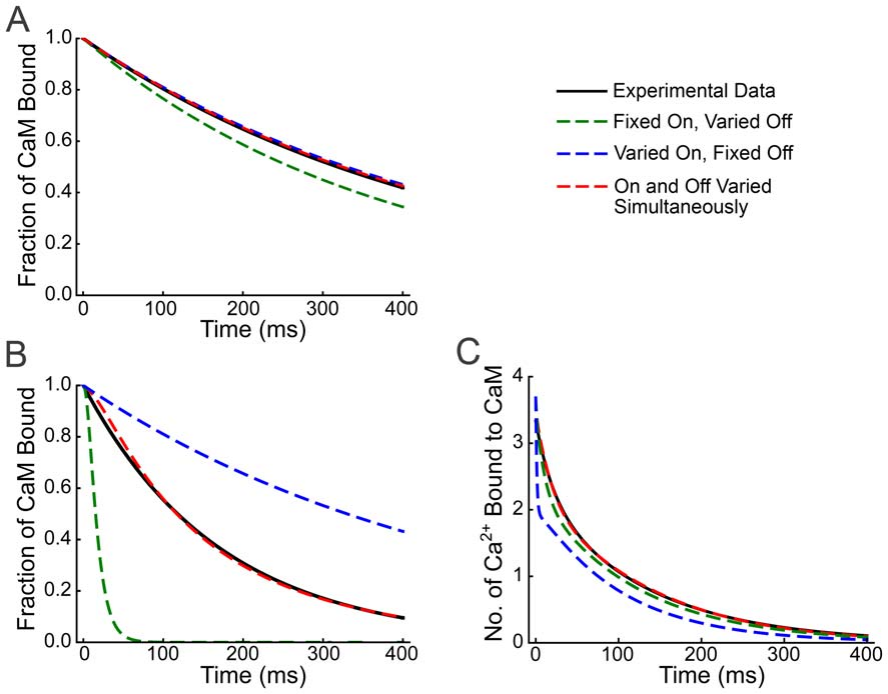
Constraining of *s* and *r* cooperativity coefficients for on and off rates by fitting to experimental data. Three independent sets of experimental data were used to constrain the values of the cooperativity coefficients *s* and *r*, that represent the ratios between the on and off binding constants for Ca^2+^ to the N- and C-termini of free CaM (respectively) and the corresponding binding constants for Ca^2+^ to the same termini in the K•CaM complex. The simplex method for gradient descent was used to fit the parameters to each set of data. A) Fits to data for dissociation of CaM from CaMKII in 50 µM Ca^2+^ (data from Figure 2B in [30]); B) Fits to data for dissociation of CaM from CaMKII in 200 nM Ca^2+^ (data from Figure 2B in [30]); and C) Fits to data for dissociation of Ca^2+^ from Ca^2+^/CaM/CaMKII (data renormalized from Figure 4A in [57]). Black, real data; Blue, best fit when all the cooperativity was assumed to reflect a change in on rates; Green, best fit when all the cooperativity was assumed to reflect a change in off rates; Red, best fit when cooperativity in on and off rates were allowed to vary simultaneously. (See Text S1 for details.)

To determine the contributions of individual on and off rates to the change in affinity of CaM for Ca^2+^ after CaM binds to mCaMKII, we define four relations: 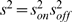, 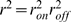, 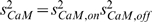 and 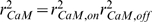). (See Text S1 for complete derivations.) The eight cooperativity coefficients (*s_on_*, *s_CaM,on_*, *r_on_*, *r_CaM,on_*, and the corresponding off coefficients), which represent four independent variables, are constrained by fitting to three sets of experimental data (Figure 4, and Text S1).

The definitions of the 8 equilibrium constants and the 47 rate constants for Model 1, and their constrained ranges of values, are given in Table S1. The fitted values of individual cooperativity coefficients are given in Table S2.

#### Model 2

**Figure 5:**
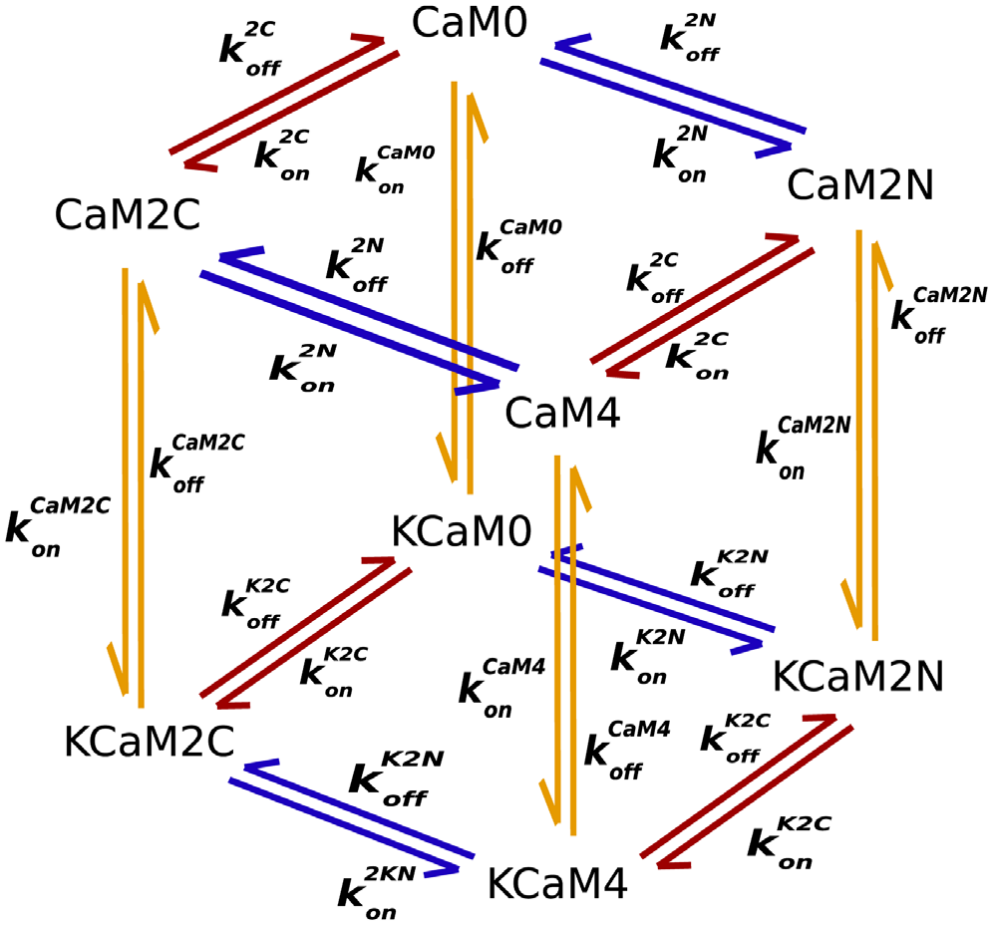
Model 2: coarse-grained model of binding among Ca^2+^, CaM and CaMKII. The reaction network includes only pairs of Ca^2+^ ions, assuming highly cooperative binding at each CaM terminus. Rate constants were derived from those for Model 1 as described in Text S1.

Model 2 (Figure 5) is a coarse-grained version of Model 1 in which we assume that association and dissociation of the two Ca^2+^ ions at each terminus occurs simultaneously. Thus, the model includes 4 distinct species of Ca^2+^/CaM; CaM0, CaM2N, CaM2C, and CaM4. The values of rate constants for Model 2 are derived directly from parameters of Model 1 as described in Text S1.

#### Model of autophosphorylation

**Figure 6:**
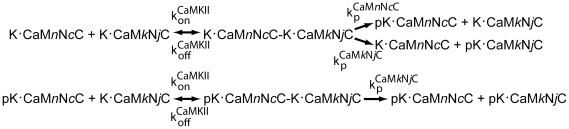
Model of autophosphorylation of one mCaMKII by another. Autophosphorylation requires that CaM be bound to both the subunit acting as “enzyme” and the subunit acting as “substrate”. A range of association rate constants for the subunit complex (Table S1) were calculated based upon estimated affinity constants from experimental studies as described in Text S1. Phosphorylated K•CaM*n*N*c*C species are denoted pK•CaM*n*N*c*C.

Autophosphorylation of mCaMKII occurs when two K•CaM*n*N*c*C species bind to form a complex, allowing one of the monomers to act as enzyme and the other as substrate (Figure 6; Hansen et al. [29]). We use the autophosphorylation model shown in Figure 6 in both Models 1 and 2. We assume that dissociation of the complex after autophosphorylation of the substrate molecule is relatively fast; thus, we do not model it explicitly. As a further simplification, we assume that, once the K•CaM*n*N*c*C-K•CaM*n*N*c*C complex forms, the autophosphorylation reaction occurs sufficiently rapidly that neither CaM nor Ca^2+^ dissociates from either kinase monomer in a complex. The intrinsic rate of autophosphorylation in a complex is ∼1 s^−1^ [25]; whereas the rate of dissociation of CaM4 from a single K•CaM4 is 1.1 to 2.3 s^−1^ [30]. Thus, our assumption is equivalent to the assumption that binding of two K•CaM*n*N*c*C species in an enzyme-substrate complex stabilizes their bound Ca^2+^/CaM. After a subunit is autophosphorylated, the off rate of CaM4 is decreased to ∼.07 s^−1^ at nM Ca^2+^ and ∼9×10^−5^ s^−1^ at µM Ca^2+^, effectively “trapping” CaM4 for several seconds, even at low Ca^2+^ concentration [30].

### Estimation of physiological concentration ranges of Ca^2+^, CaM, and CaMKII

Many of the simulations were carried out with concentrations of Ca^2+^, CaM, and CaMKII that approximate those in postsynaptic spines of excitatory neurons in the forebrain. The concentration ranges of CaM, CaMKII, and Ca^2+^ in spines were estimated from previous biochemical studies as follows. The average protein concentration in rat brain was taken as 100 mg/ml (equivalent to ∼10% by weight, see [31]). CaMKII is an unusually abundant enzyme in the forebrain; its concentration is 2% of total protein by weight in the hippocampus and 1% in the rest of the forebrain as measured by quantitative immunoblot [32]. Therefore, its average concentration in the hippocampus is ∼2 mg/ml. CaMKII is found almost entirely in excitatory neurons which account for approximately half of forebrain weight, the rest consisting of inhibitory neurons, glial cells, blood vessels, and other minor cell types. Thus, the average concentration of CaMKII in excitatory neurons is ∼4 mg/ml. Given that the molecular weight of individual CaMKII subunits is ∼56 kDa, the average concentration of CaMKII catalytic subunits in the hippocampus is ∼74 µM. In the rest of the forebrain, the average concentration is ∼37 µM. Several studies have shown that CaMKII is usually more concentrated in the heads of spines than in dendritic shafts [e.g. 33] and is highly concentrated in the postsynaptic density fraction [34]. On the other hand, CaMKII appears to move into or out of spines in response to synaptic activation [35],[36] and can associate with proteins in or near the PSD [7]. Thus, in our simulations, we explore the effect of concentrations of CaMKII subunits from 40 to 200 µM on the rate of autophosphorylation. When studying other variables, we set the concentration of mCaMKII at 80 µM.

The concentration of CaM in bovine and rat brain varies from ∼17 µM in the hippocampus [3] to ∼26 µM in the cerebral cortex and whole brain [3],[4]. If CaM in the particulate fraction is included, the estimated concentration in brain rises to ∼33 µM [3]. In our simulations, we use concentrations of CaM from 20 to 40 µM.

The concentration of Ca^2+^ in postsynaptic spines varies dramatically. Basal concentrations from 50 to 500 nM have been reported; whereas, in the immediate vicinity of activated NMDA receptors in the PSD, the transient concentration can rise to tens of µM [28], [37]–[39]. Here, we explore the dynamics of autophosphorylation of CaMKII through a large range of physiological Ca^2+^ concentrations and in response to trains of brief calcium transients (msec duration), similar to those thought to occur in neurons. We also examine autophosphorylation at steady-state concentrations of Ca^2+^ ranging from 0.5 to 250 µM, which mimic experimental conditions. In all simulations, the concentration of Mg^2+^-ATP is assumed to be saturating, as it would be in the cell.

### Simulation methods

Reaction networks were entered into Mathematica [40] with the Xcellerator package [41] which translated the networks into systems of ODEs based on the law of mass action. Numerical integration was performed in Mathematica [40]. This method assumes well-mixed conditions and thus only approximates the situation in the cytosol.

Ca^2+^ spikes were simulated as exponential functions 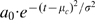; where 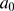 and 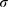 set the spike height and half width, respectively; 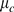 centers the spike at a location relative to the last spike, depending upon the input frequency; and *t* is time. This function was used as a fixed boundary condition representing the free Ca^2+^ concentration. Thus, total Ca^2+^ was not conserved over the sum of the driving function and the Ca^2+^ bound to various molecular species. This algorithm simulates a neuronal environment in which Ca^2+^ enters the cytosol through voltage and ligand-gated channels and is then rapidly sequestered or removed. Mathematica packages implementing the models are available from the authors.

### Sensitivity analysis

We used sensitivity analyses to determine which parameters of Model 1 (Table S1) produce the most variation in the predicted autophosphorylation of mCaMKII. We assembled random sets of input parameters, sampled over the range of experimental values for each parameter, using Latin Hypercube sampling [42]–[44], as described in Text S1. The values were taken from Table S1, and from the range of estimates of physiological concentrations of Ca^2+^, CaM, and mCaMKII (above). We then calculated output of the model for each set of randomized parameters every 0.05 s for a 2 s simulation. In one set of calculations, we used a series of fixed concentrations of Ca^2+^ to examine how the importance of individual parameters varies at different Ca^2+^ concentrations. The contribution of variations in each input parameter to variation in the output was quantified by calculating the partial rank correlation coefficient (PRCC) [42]–[45],[reviewed in 46], as described in [47]. The PRCC quantifies the correlation of values of each individual parameter with the output, when the linear effects of the other parameters on output are removed. A perfect positive correlation gives a PRCC of 1; whereas, a perfect negative correlation gives a PRCC of −1. Details are described in Text S1.

## Results

### Time evolution of Ca^2+^ binding to CaM and its effect on autophosphorylation

**Figure 7:**
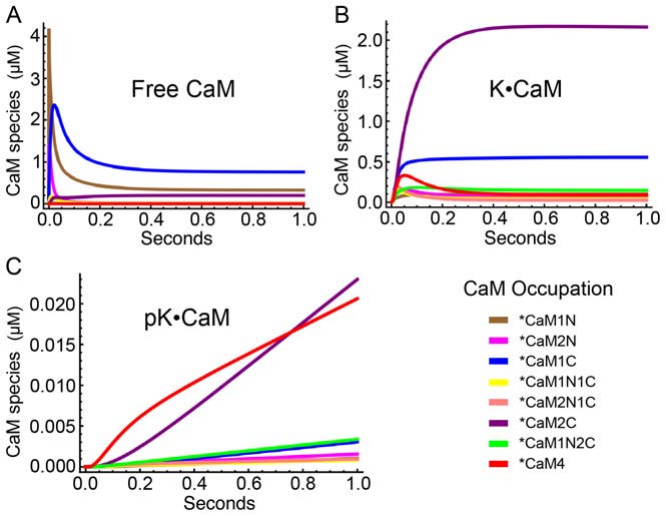
Time courses of species of CaM, K•CaM, and pK•CaM with varying numbers of bound Ca^2+^ ions, simulated with Model 1. The initial conditions for the simulation were [CaM] = 30 µM, [mCaMKII] = 80 µM, and [Ca^2+^] = 10 µM. A) Time course of formation of species of free CaM. B) Time course of formation of species of CaM bound to CaMKII (K•CaM). C) Time course of formation of species of CaM bound to phosphorylated CaMKII (pK•CaM). The color code for Ca^2+^ occupation of sites on CaM is indicated on the lower left. *color code applies to all forms of CaM with the indicated bound Ca^2+^. Note differences in scale for panels A), B) and C).

Model 1 was used to predict the time evolution of binding of Ca^2+^ to CaM and of Ca^2+^/CaM to mCaMKII after a rapid increase in concentration of Ca^2+^. In particular, we examined the time evolution when the concentrations of Ca^2+^ or CaM are not high enough to saturate binding to mCaMKII; conditions that are likely to prevail in postsynaptic spines during activation of NMDARs [7]. Figure 7 shows the predicted time evolution of all CaM species (0–4 bound Ca^2+^ ions) when 10 µM Ca^2+^ was introduced at time zero into a system containing 30 µM CaM and 80 µM mCaMKII. We picked these values because they are within the likely physiological ranges of concentrations in a spine during activation of NMDARs. Changes in the species of free CaM (Figure 7A), CaM bound to mCaMKII (Figure 7B), and CaM bound to autophosphorylated mCaMKII (Figure 7C) were plotted during one sec of simulation. Ca^2+^ bound rapidly to the N-terminus of free CaM within the first few msecs after addition, resulting in peaks in the concentrations of CaM1N and CaM2N (Figure 7A; brown and pink). Because Ca^2+^ also dissociates rapidly from these sites, the concentrations decayed within the first 200 msec to a relatively low equilibrium value. In contrast, Ca^2+^ bound more slowly to the C-terminus of CaM (blue and purple), but free CaM1C (blue) reached a relatively high equilibrium concentration because Ca^2+^ has a higher affinity for the C-terminal sites. The equilibrium concentration of free CaM2C remained low because this species (purple) binds very rapidly to mCaMKII (9.2 µM^−1^ sec^−1^, see Table S1). Thus, by 50 msecs, K•CaM2C was the most abundant K•CaM species in the simulation (Figure 7B, purple).

We have shown experimentally that CaM2C and CaM2N support autophosphorylation upon binding to mCaMKII, although at a rate 10 to 20-fold lower than CaM4 [25]. Because of the faster autophosphorylation rate of CaM4, the most abundant autophosphorylated mCaMKII species throughout most of the simulation was pK•CaM4 (Figure 7C, red). Nevertheless, under the conditions of this simulation in which the concentrations of Ca^2+^ and CaM are limiting, pK•CaM2C became the dominant species by the final 200 msecs (Figure 7C, purple).

To test whether the actual binding of mCaMKII to CaM species with less than 4 bound Ca^2+^ ions influenced the final extent of autophosphorylation under these conditions, we constrained Model 1 such that only CaM4 could bind directly to mCaMKII. Thus, we set reaction rates to zero for all the vertical yellow arrows in Figure 2C, except the on and off rates for binding of CaM4 to mCaMKII to form K•CaM4. We altered the model for autophosphorylation such that only K•CaM4 could be autophosphorylated. However, we continued to allow K•CaM species with less than four bound Ca^2+^ ions to carry out autophosphorylation of K•CaM4 as follows:
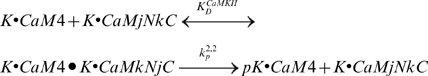
Thus, we continued to allow dissociation reactions in which K•CaM4 loses Ca^2+^ ions. However, as in the complete Model 1, we assumed that after autophosphorylation, pK•CaM4 did not lose either CaM or its bound Ca^2+^ during a one sec reaction.

**Figure 8:**
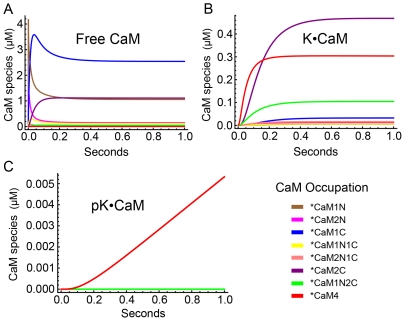
Time courses of species of CaM, K•CaM, and pK•CaM with varying numbers of bound Ca^2+^ ions, simulated with Model 1 altered to allow binding of only CaM4 to CaMKII, and autophosphorylation of only K•CaM4. The initial conditions were as in Figure 7. A) Time course of formation of species of free CaM. B) Time course of formation of species of CaM bound to CaMKII (K•CaM). C) Time course of formation of species of CaM bound to phosphorylated CaMKII (pK•CaM). The level of pK•CaM4 after 1 sec is 3 times lower than in the simulation with the complete Model 1 (Figure 7C). This demonstrates that the dominant pathway to pK•CaM4 at short times under these conditions is via Ca^2+^ binding to K•CaM species with fewer than 4 bound Ca^2+^ ions. The color code for Ca^2+^ occupation of sites on CaM is indicated on the lower left. *color code applies to all forms of CaM with the indicated bound Ca^2+^. Note differences in scale for panels A), B) and C).

Figure 8 shows the time evolution of all CaM species predicted by this limited model under the same conditions as in Figure 7. The time evolution of free CaM species (Figure 8A) was similar to that in Figure 7A, although free CaM with less than 4 bound Ca^2+^ ions reached higher equilibrium concentrations, presumably because they could not bind to mCaMKII. A larger divergence between the full and limited Models is evident in Figure 8B. The total concentration of CaM species bound to mCaMKII after one sec was reduced from ∼3 µM in Figure 7B to less than 1 µM in Figure 8B (note difference in scales of the ordinates). Conversely, the concentration of K•CaM4 (Figure 8B, red) was elevated relative to the other K•CaM species presumably because the nonsaturated CaM species could not bind directly to mCaMKII, leaving more of them to bind Ca^2+^ and be “promoted” to free CaM4, after which they could bind to mCaMKII. The total concentration of all K•CaM species with fewer than 4 bound Ca^2+^ was considerably reduced compared to Figure 7B because the only kinetic pathway by which these species could be formed was via loss of Ca^2+^ from K•CaM4. The only phosphorylated species was pK•CaM4 (Figure 8C), as dictated by the design of the limited Model 1. The most interesting result was that the equilibrium concentration of pK•CaM4 species in the limited model was only ∼25% of the level reached in the full model (∼0.02 in Figure 7C vs. ∼0.005 in Figure 8C; note difference in scale of the ordinates). This result means that, when concentrations of Ca^2+^ and CaM are limiting, the most important pathway toward formation of pK•CaM4 in Model 1 is via binding of Ca^2+^ to partially filled K•CaM species prior to autophosphorylation. Thus, even if autophosphorylation of K•CaM2C and K•CaM2N could not occur, these partially filled CaM species would assume an important kinetic role in the autophosphorylation reaction, presumably because binding of CaM to the kinase target enhances the affinity of CaM for Ca^2+^.

This kinetic pathway may have general significance for signaling through CaM because theoretical considerations suggest that different targets of Ca^2+^/CaM have different abilities to stabilize Ca^2+^/CaM species, depending on the structures of their CaM binding sites and surrounding residues. The kinetic role of stabilization of sub-saturated Ca^2+^/CaM species by targets may significantly influence the outcome of regulatory events initiated by Ca^2+^ transients *in vivo*; and these outcomes may not be accurately predicted by the behavior of the enzyme targets at saturating, steady-state Ca^2+^/CaM concentrations in a test tube.

### Comparison of Model 1 to simpler models of interaction among Ca^2+^, CaM, and mCaMKII

**Figure 9:**
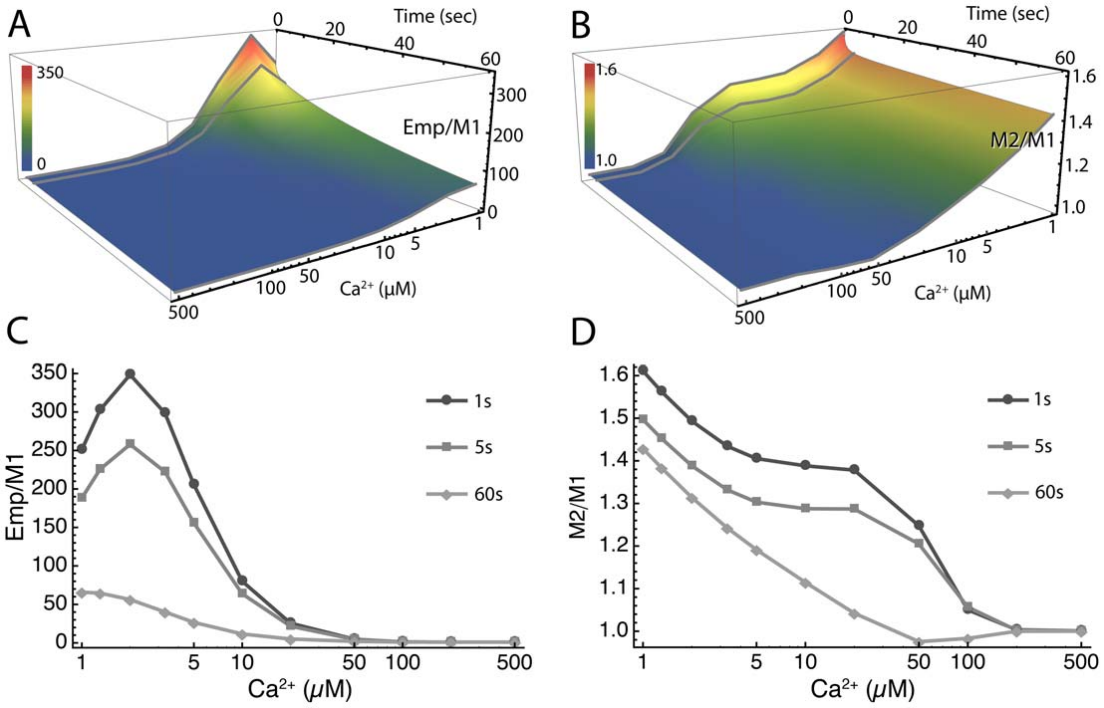
Differences in predicted autophosphorylation between Model 1, Model 2, and an Empirical Model, at varying concentrations of Ca^2+^ and reaction times. A) Surface plot of ratio of autophosphorylation predicted by the Empirical model and by Model 1. B) Surface plot of ratio of autophosphorylation predicted by Model 2 and by Model 1. In A and B Contour lines for 1, 5, and 60 sec reaction times are shown in light gray. C) Ca^2+^-dependence of the ratio of autophosphorylation predicted by the Empirical model and Model 1 at 1, 5, and 60 sec reaction times. D) Same as C for ratio of autophosphorylation predicted by Model 2 and Model 1.

We compared predictions of Model 1 to two other models of interactions of Ca^2+^, CaM, and CaMKII: Model 2, a coarse-grained 4 state model derived from Model 1 (Figure 5) and the “Empirical” Model, a 2 state model in which only CaM4 can bind to CaMKII. The Empirical Model includes a version of the Adair-Klotz equation which represents the relation between Ca^2+^ concentration and levels of CaM4 [48], and assumes cooperativity of binding of the four Ca^2+^ ions to CaM. This empirical model is similar to other 2-state models that have been used in studies of CaMKII [49],[50]. The initial concentrations of free CaM and mCaMKII were set to 30 µM and 80 µM, respectively, as in Figures 7 and 8, and ratios of the output of these two models to that of Model 1 were calculated, varying Ca^2+^ from 1 to 500 µM, and time from 0 to 60 sec (Figure 9). The output of Model 2 differs considerably from Model 1 at physiological concentrations of Ca^2+^ (1 to 30 µM). This result means that Model 1 is required to obtain the most acccurate estimates of binding of Ca^2+^ to CaM and CaMKII under the conditions that prevail in a spine; the simpler Model 2 can be used when accuracy within a factor of 2 is adequate. In contrast, the differences between the empirical model and Model 1 in the same physiological range of Ca^2+^ are much greater. For example, at 10 µM Ca^2+^, the empirical model predicts ∼100 fold higher autophosphorylation after 1 sec than does Model 1; whereas, Model 2 predicts ∼1.12 fold higher autophosphorylation than does Model 1. This result means that the empirical model, and by inference other 2 state models, do not accurately predict Ca^2+^/CaM dynamics at concentrations of Ca^2+^, CaM, and CaMKII present *in vivo*. Thus, in order to achieve the highest accuracy in predictions of CaMKII activity in a spine, it is necessary to include the kinetic details of binding among Ca^2+^, CaM, and CaMKII.

### The kinetics of interaction among Ca^2+^, CaM, and mCaMKII produce frequency dependence of autophosphorylation during transient Ca^2+^ signals

Previous investigators have studied the dependence of activation of CaMKII on the frequency of rapid series of Ca^2+^ transients such as occur inside a cell during signaling. One experimental study demonstrated a frequency dependence by applying brief pulses (80 ms to 1 s at 0.1 to 10 Hz) of fully saturated Ca^2+^/CaM (500 µM Ca^2+^, 0.1 to 0.4 µM CaM) to immobilized CaMKII and then measuring the resulting Ca^2+^-independent catalytic activity [51]. Autophosphorylation was dependent on frequency between ∼0.5 and 4 Hz for 100 ms pulses, and between ∼2 and 10 Hz for 80 ms pulses. The authors theorized that the frequency dependence arises from the requirement that two CaM's must bind to two adjacent kinase subunits in a holoenzyme to initiate autophosphorylation [29]. Thus, if the off rate for dissociation of Ca^2+^/CaM from a single subunit is significantly slower than the inter-stimulus interval of the Ca^2+^ transients, some Ca^2+^/CaM will remain bound individual subunits and contribute to activation of autophosphorylation during the next transient stimulus. The theoretical model of Kubota and Bower, which included the empirical model described in Figure 9A for association of Ca^2+^, CaM, and CaMKII, also supported this same mechanism [48]. We found that Model 1 predicts an additional mechanism for frequency dependence in which the kinetics of Ca^2+^ binding to the C terminus of CaM in the K•CaM complex give rise to frequency dependence of autophosphorylation in the 1 to 8 Hz range.

**Figure 10 pcbi-1000675-g010:**
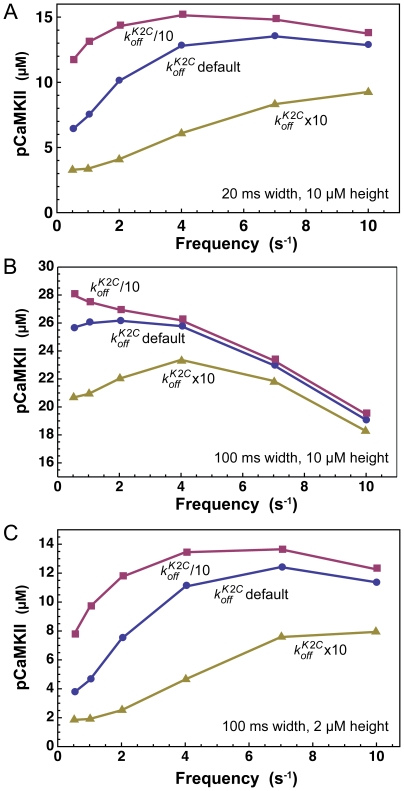
Frequency dependence of autophosphorylation produced by Ca^2+^ binding dynamics. Simulations were performed with [CaM] = 30 µM and [mCaMKII] = 80 µM. Each line plots summed autophosphorylation of all kinase complexes in response to a series of 30 Ca^2+^ spikes at varying frequencies simulated with Model 1 as described in Methods. A) The half width of each spike (*σ* ) is set to 10 ms (width = 20 ms, FWHM = 16 ms) and the peak height (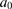) is 10 µM. B) The half width of each spike (*σ* ) is set to 50 ms (width = 100 ms, FWHM = 83 ms) and the peak height (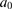) is 10 µM. C) The half width of each spike (*σ* ) is set to 50 ms (width = 100 ms, FWHM = 83 ms) and the peak height (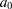) is 2 µM. Blue, simulations with all parameters set to default (midpoint of ranges in Table S1). Gold, same as blue except that the default 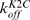 is multiplied by 10 to produce faster decay of K•CaM2C. Magenta, same as blue except that the default 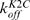 is divided by 10 to produce slower decay of K•CaM2C.

Figure 10 shows plots of summed autophosphorylation after 30 Ca^2+^ pulses, as a function of frequency of the pulses. Figure 10A illustrates pulses of width 20 ms; Figures 10B and C, pulses of 100 ms. The three curves in each figure were generated with three different values of 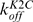; default (median of range in Table S1, blue), default divided by 10 (magenta), and default times 10 (yellow). The default value produces 2-fold variation in autophosphorylation from 0.5 to 4 Hz for 20 ms pulses of height 10 µM, no frequency dependence for 100 ms pulses of 100 µM, and a 3-fold variation from 0.5 to 7 Hz for 100 ms pulses of height 2 µM. Faster values of 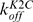 decrease the range and magnitude of frequency dependence; whereas, slower values increase the range of the frequency dependence.

To determine whether this form of frequency dependence requires that two CaM's must bind to two kinase monomers to initiate autophosphorylation, Model 1 was altered to permit zero-order autophosphorylation (that is autophosphorylation without the requirement for monomer-monomer interactions). The modified model showed similar frequency dependence (data not shown), indicating that the requirement for two CaM's binding to two monomers does not play a large role in this mechanism of frequency dependence.

**Figure 11:**
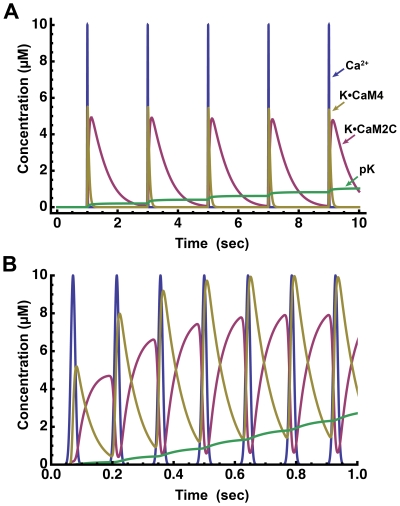
Interaction of time evolution of K•CaM2C and K•CaM4 with frequency of Ca^2+^ pulses. Simulations were performed with [CaM] = 30 µM and [mCaMKII] = 80 µM. Time courses of Ca^2+^ (blue), K•CaM2C (magenta), K•CaM4 (gold) and summed autophosphorylated CaMKII (green) are plotted. A) Pulses with half width (σ) set to 10 ms (width = 20 ms, FWHM = 16 ms) and peak height (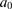) set to 10 µM were simulated at 0.5 Hz for 10 sec. B) Same as A) but frequency of 7 Hz was simulated for 1 sec. All parameters were set to default as in Figure 10, blue lines.

To explore the mechanism further, we examined how the frequency of Ca^2+^ pulses affects the accumulation of CaM species during each pulse. Ten and 1 sec of 20 ms pulses of 10 µM Ca^2+^ were simulated at 0.5 and 7 Hz, respectively. At 0.5 Hz (Figure 11A), K•CaM2C and K•CaM4 formed by a single pulse dissociated completely before the next pulse began. Thus, there was no interaction between the species formed from one pulse to the next, and no frequency dependence of autophosphorylation. In contrast, at 7 Hz, K•CaM2C was entirely converted to K•CaM4 during each pulse, but some of the K•CaM4 dissociated into K•CaM2C during the inter-pulse interval. The additional K•CaM2C was converted to K•CaM4 during the next pulse. Thus, the concentrations of K•CaM2C and K•CaM4 increased significantly with each pulse, resulting in a slightly higher level of autophosphorylation after the same number of pulses at 7 Hz, compared to 0.5 Hz. This small increase translates into a 2-fold increase in autophosphorylation for 30 pulses at 7 Hz compared to 30 pulses at 0.5 Hz (Figure 10A).

### Sensitivity of autophosphorylation to variations in input parameters for Model 1

We performed sensitivity analyses, as described under Methods, to identify which parameters most influence the outcome of Model 1. The analyses were carried out in two different ways. We first examined the importance of each input parameter based on the range of the estimated experimental uncertainty in its measurement, as listed in Table S1. For this analysis, parameters were varied over the full range of values in Table S1. Values of parameters that do not have ranges, or for which the range is unknown, were varied 4-fold with the value in Table S1 taken as the mean. We next determined the importance of each parameter without using the estimated range of experimental uncertainty. For that analysis, we assumed that the mean values are accurate estimates of the real mean. Parameters were varied 2.5-fold around the mean values in Table S1. This second analysis measured the influence of the relative magnitude of each parameter and its position in the model rather than the limitations of experimental estimates of individual parameters.

We used PRCC values (calculated as described under Methods) to describe the relative importance of each parameter for predicting the level of autophosphorylation. Not surprisingly, we found that autophosphorylation is highly sensitive to changes in Ca^2+^ concentration when all parameters are varied globally (Table 1). Because Ca^2+^ signaling *in vivo* often occurs over a period of a few hundred msecs or less, we examined which parameters most influence autophosphorylation levels at different times during a reaction. We calculated PRCC's for time series under three different regimes of Ca^2+^ concentration; low (1–5 µM), medium (10–50 µM), and high (50 to 250 µM). The low regime encompasses the range believed to arise in and near the PSD during low frequency stimulation of NMDA-receptors [28],[52]. The medium regime encompasses the concentrations believed to occur in the PSD during strong stimulation of NMDA receptors [28],[52]. Concentrations above 100 µM likely do not occur under normal physiological conditions, but are frequently used in enzymatic experiments in the laboratory. As expected, the importance of specific binding parameters varies considerably with time and among the three Ca^2+^ regimes. Table 1 lists the parameters having a PRCC value either above 0.3 or below −0.3, indicating a strong correlation (positive or negative, respectively) with the output value.

**Table 1:** The sensitivity of phosphorylation of CaMKII to variations in input parameters as measured by partial rank correlation coefficient (PRCC) at different Ca^2+^ concentration ranges.

0.1 sec			1 sec			2 sec		
Parameter	Confidence Interval		Parameter	Confidence Interval		Parameter	Confidence Interval	
**Low Ca^2+^ (1–5 µM)**								
[Ca^2+^]	0.77	0.72	[Ca^2+^]	0.77	0.72	[Ca^2+^]	0.77	0.72
[CaMKII]	0.73	0.66	[CaMKII]	0.70	0.64	[CaMKII]	0.70	0.63
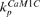	0.49	0.39	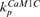	0.61	0.53	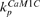	0.62	0.54
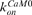	0.47	0.37	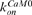	0.51	0.41	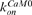	0.51	0.41
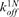	0.36	0.25	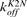	−0.25	−0.36	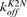	−0.24	−0.35
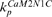	0.35	0.24	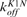	−0.26	−0.37	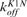	−0.26	−0.37
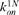	−0.25	−0.36	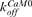	−0.44	−0.53	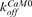	−0.48	−0.57
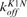	−0.26	−0.37	K_D_	−0.68	−0.75	K_D_	−0.68	−0.75
K_D_	−0.67	−0.74						
**Medium Ca^2+^ (10–50 µM)**								
[Ca^2+^]	0.78	0.73	[Ca^2+^]	0.78	0.73	[Ca^2+^]	0.78	0.73
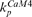	0.67	0.59	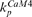	0.57	0.47	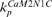	0.55	0.45
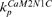	0.54	0.45	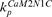	0.55	0.46	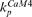	0.53	0.43
[CaMKII]	0.47	0.36	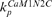	0.47	0.36	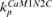	0.47	0.37
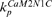	0.39	0.27	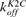	0.42	0.31	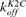	0.46	0.36
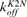	−0.30	−0.41	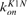	0.41	0.31	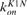	0.42	0.31
[CaM]	−0.36	−0.46	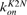	0.38	0.27	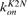	0.37	0.26
K_D_	−0.67	−0.74	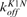	−0.31	−0.42	[CaM]	−0.30	−0.40
			[CaM]	−0.32	−0.42	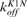	−0.31	−0.42
			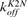	−0.37	−0.48	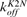	−0.36	−0.46
			K_D_	−0.67	−0.73	K_D_	−0.67	−0.73
**High Ca^2+^ (50–250 µM)**								
[Ca^2+^]	0.70	0.63	[Ca^2+^]	0.68	0.61	[Ca^2+^]	0.68	0.60
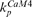	0.64	0.56	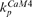	0.59	0.50	[CaM]	0.57	0.48
[CaM]	0.54	0.45	[CaM]	0.54	0.45	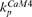	0.55	0.46
K_D_	−0.53	−0.61	K_D_	−0.46	−0.55	K_D_	−0.42	−0.51

Ranges of parameters other than [Ca^2+^] set as in Table S1. Model parameters are ranked by their PRCC values at 0.1, 1 and 2 seconds after Ca^2+^ addition. Parameters with PRCC values lower than 0.3 are not shown.

The concentration of CaMKII subunits was an important determinant at low Ca^2+^; whereas the concentration of CaM assumed more importance at higher Ca^2+^ and longer times. The K_D_ for the interaction between two CaMKII subunits with bound CaM was a strong determinant of the output at all Ca^2+^ concentrations and times (Table 1).

Of the 47 individual rate constants, 14 had a significant PRCC value in at least one of the regimes. In low Ca^2+^, 6 rate constants at 0.1 sec, and 5 at 1 and 2 secs, had significant PRCCs; in medium Ca^2+^, 4 at 0.1 sec, and 8 at 1 and 2 secs, had significant PRCC's; and in high Ca^2+^, only the intrinsic rate of autophosphorylation had a significant PRCC.

At lower Ca^2+^ concentrations and shorter times, the most important rate constants are those for formation of K•CaM species with fewer than 4 bound Ca^2+^; and the autophosphorylation rate constants, 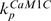 and 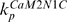. When the narrower range of parameters is used in the calculations (Table S3), 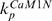 and 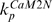 replace 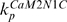. Thus, the ability of K•CaM complexes with few bound Ca^2+^ ions to support autophosphorylation is critical at low Ca^2+^. At medium Ca^2+^, K•CaM4 has a strong influence on autophosphorylation at all times because its autophosphorylation rate constant ( 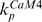 ) is 10 times higher than that of K•CaM2C (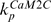). The rate constants for binding of Ca^2+^ to K•CaM at the N terminus (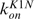, 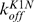, 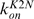, 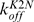) have a strong influence, reflecting the fact that K•CaM2C reaches a higher concentration than K•CaM2N after the first 100 msecs because of the higher affinity of the C-terminus for Ca^2+^. Thus, the rate of conversion of K•CaM2C to K•CaM4 by binding of Ca^2+^ to the N-terminus of CaM is critical. In the high Ca^2+^ regime, which represents the usual well-mixed experimental conditions, Ca^2+^ concentration, 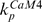, CaM concentration, and the K_D_ for monomer-monomer association are the determining parameters.

**Figure 12:**
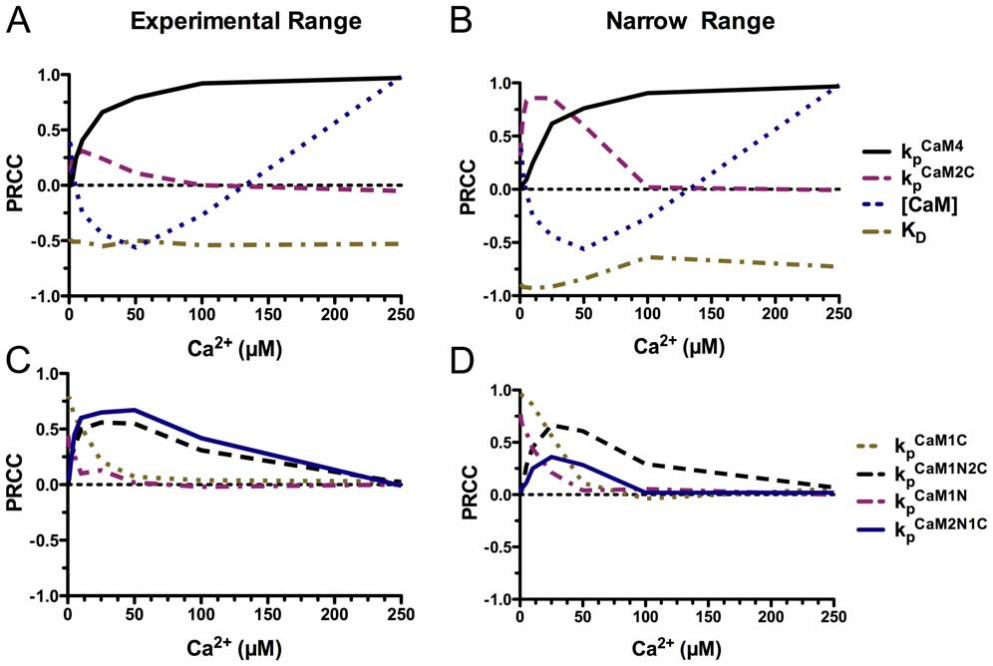
Variation in PRCC of selected parameters as a function of Ca^2+^ concentration. PRCC values for a 1 sec reaction are plotted as a function of initial free Ca^2+^ concentration. A) PRCC values were calculated using the range of experimental uncertainty in parameter values from Table S1. B) PRCC values were calculated as in panel A, except that more restricted ranges of parameter values were used (2.5-fold around the mean values in Table S1). C) PRCC values for the autophosphorylation rates of K•CaM complexes with odd numbers of bound Ca^2+^ were calculated as in panel A. D) PRCC values for the autophosphorylation rates of K•CaM complexes with odd numbers of bound Ca^2+^ were calculated as in panel B.

Figure 12 A and B illustrate that the importance of some of the parameters varies dramatically with Ca^2+^ concentration during a 1 s reaction. Interestingly, the concentration of CaM is inversely correlated with autophosphorylation between ∼20 and ∼100 µM Ca^2+^. Partially bound Ca^2+^/CaM species are more prevalent than fully bound CaM4 at these Ca^2+^ concentrations. Thus, higher CaM concentrations may result in less autophosphorylation because binding to extra CaM reduces the amount of Ca^2+^ available for binding to K•CaM species.

Figures 12 B and C also illustrate the differing importance of the intrinsic autophosphorylation rate constants for K•CaM2C and K•CaM4 (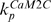 and 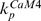, respectively) as the Ca^2+^ concentration rises. Below ∼25 µM Ca^2+^, the two species have approximately equal influence on autophosphorylation. However, above 100 µM Ca^2+^, K•CaM4 and its autophosphorylation rate constant dominate. The differences between the PRCC curves for autophosphorylation rate constants in Figures 12 C and D reflect the influence of K•CaM species with odd numbers of bound Ca^2+^ (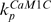, 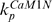, 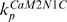, 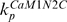), which have very high experimental uncertainty. For example, note how the influence of 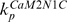 decreases when the range of uncertainty is narrowed in Figure 12D. The experimental range of values for 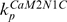 in Figure 12C (Table S1) spanned the measured value for 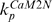 (0.079 s^−1^) to that for 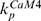 (1.25 s^−1^), a range of 16-fold. In contrast, the narrower range of values (Figure 12D) spanned 0.38 s^−1^ to .95 s^−1^, a range of 2.5 fold around the mean of 0.66 s^−1^. The importance of 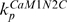, which has a smaller experimental variability than 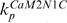, increased slightly from Figure 12C to 12D; whereas, those of 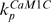 and 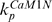 both increased significantly at the lower Ca^2+^ concentrations.

### Important parameter values that can be specified more precisely by improved experimental measurement

Several of the parameters for Model 1 have been measured relatively accurately. For example, the macroscopic binding constants for binding of four Ca^2+^ ions to CaM have been measured by several laboratories [16],[49],[53]. We calculated the microscopic constants from the macroscopic constants by solving a system of algebraic equations (see Text S1). The autophosphorylation rates 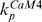, 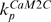 and 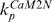 [25]; the binding and dissociation rates for CaM4 to mCaMKII (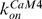, 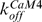); and the affinities (K_act_'s) of CaM2C and CaM2N for CaMKII (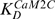, 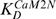) have all been measured [25]. In contrast, most of the parameters for binding of CaM to mCaMKII, binding of Ca^2+^ to K•CaM, and autophosphorylation of K•CaM species with fewer than 4 bound Ca^2+^ have not been measured and were derived, or deduced from fits of the model to experimental data in the literature, as described in Methods.

We have used global sensitivity analysis to identify parameters that have a strong impact on autophosphorylation of mCaMKII in particular concentration regimes. Thus, we have identified which of the relatively uncertain parameters will be most important to measure experimentally in the future. One of the least well defined parameters is the affinity of CaMKII monomers for each other in the autophosphorylation reaction (K_D_). The lowest estimate of the dissociation constant (highest affinity) was given by Hanson and Schulman (1994) as 1.3 µM. However, a number of other studies suggest that the affinity is considerably lower (K_D_≈20–40 µM) [54]. Given the large possible range it is not surprising that we consistently find that autophosphorylation is sensitive to this parameter (Table 1 and Figures 9–11).

In general, the parameters that describe binding of CaM to CaMKII do not have a strong influence on autophosphorylation. One exception is binding of CaM0 to CaMKII at low Ca^2+^ concentration (Table 1). At low Ca^2+^, binding of CaM to CaMKII increases the affinity of Ca^2+^ for K•CaM relative to CaM [5]; thus, facilitating the binding of Ca^2+^ to CaM and indirectly increasing the rate of autophosphorylation. In low Ca^2+^, CaM0 is the predominant species. Therefore, even though its affinity for CaMKII is low, the concentration of K•CaM0 is significantly greater than that of other K•CaM species. It will be especially important for the accuracy of Model 1 to directly measure the affinity of two K•CaM subunits for each other and the affinity of free CaM with no bound Ca^2+^ (CaM0) for CaMKII.

## Discussion

We have constructed a kinetic model of interactions of Ca^2+^, CaM, and monomeric subunits of CaMKII that can be used to understand the dynamics of activation of CaMKII by Ca^2+^ in the environment of a postsynaptic glutamatergic spine. Activation of CaMKII by Ca^2+^ flowing through NMDA-type glutamate receptors is a critical early step in synaptic changes that underlie learning and memory. We constructed this model to represent binding of Ca^2+^ and CaM to monomeric subunits, rather than to the dodecameric holoenzyme, so that it can be used to experimentally test and verify parameters for activation of CaMKII in the absence of cooperativity of CaM binding caused by the structure of the holoenzyme [26]. Thus, we can use it to eliminate ambiguity in experimental measurements of parameters. As a first step, where possible, we assigned values of parameters based on experimental measurements in our own laboratory and from the literature. When direct measurements were not available, we derived values from experimental data using conservative assumptions. In a few cases, the uncertainty in the values of parameters is large and we have shown by parameter variation and sensitivity analysis that the accuracy of the model will benefit from more precise measurements of those parameters, including the affinity of two K•CaM subunits for each other and the affinity of CaM0 for CaMKII.

An important finding is that two types of models, which are more coarse-grained than our Model 1, produced significantly different predictions for rates of autophosphorylation in a physiological regime; in particular, when simulations were run for times shorter than ∼5 sec, and at Ca^2+^ concentrations lower than ∼50 µM, with concentrations of CaM and CaMKII set to approximate those present in the spine. We conclude that these coarse-grained models are inadequate to predict the timing and extent of activation of CaMKII under physiological conditions because they do not capture critical aspects of the dynamics in the physiological regime. For example, a model that treats the binding of two Ca^2+^ ions at the amino- or carboxyl-termini of CaM as simultaneous (Model 2), overestimates the rate of autophosphorylation by 30 to 60% under these conditions, compared to Model 1. An Empirical model which is similar to those often used in previous models of activation of CaMKII [48] overestimates the rate of autophosphorylation 100 to 350-fold in the physiological regime (Figure 9).

**Figure 13:**
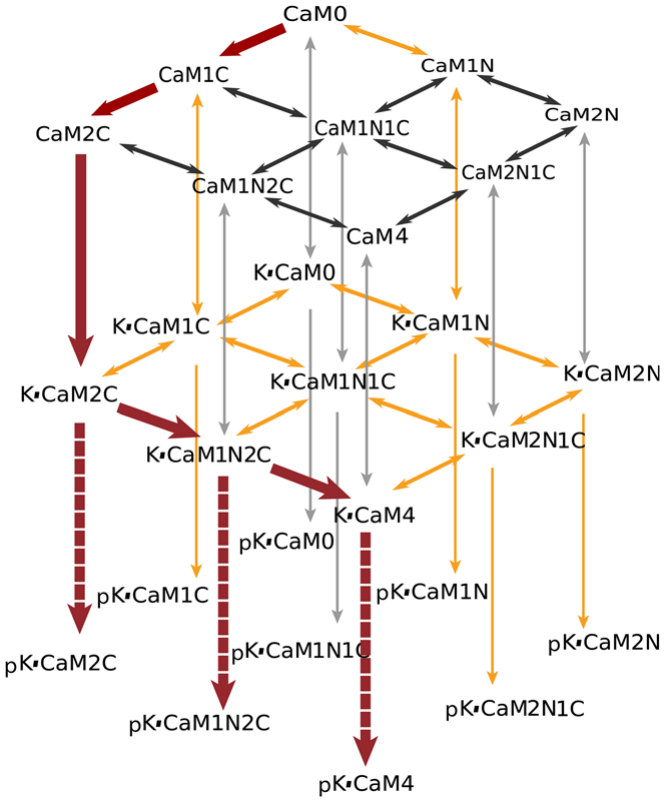
Hypothetical kinetic pathways leading to autophosphorylation of CaMKII. Paths shown in yellow are significant at Ca^2+^ concentrations below ∼30 µM and at times up to 1 sec after an increase in Ca^2+^ concentration. Paths shown in red predominate at Ca^2+^ concentrations above ∼30 µM.

A second key finding is that species of CaM with fewer than 4 bound Ca^2+^ ions are major activators of CaMKII at Ca^2+^ concentrations as high as ∼30 µM, a concentration that falls in the middle of the physiological regime. Simulations of formation of CaM species such as those shown in Figure 7, together with parameter sensitivity analyses (Figure 12 and Table 1) suggest that the major kinetic pathways through which Ca^2+^ binds to CaM, and Ca^2+^/CaM binds to and activates CaMKII, differ during the first sec of Ca^2+^ influx, depending on the peak Ca^2+^ concentration (Figure 13). Below ∼30 µM Ca^2+^, K•CaM2C plays a significant role as a precursor of autophosphorylated kinase. Furthermore, because the affinity of CaM for Ca^2+^ is significantly increased when it binds to CaMKII, the kinetic pathways involving direct binding of Ca^2+^ to K•CaM (yellow in Figure 13) are more significant than those in which Ca^2+^ binds first to free CaM. At concentrations of Ca^2+^ greater than ∼30–50 µM, CaM2C and K•CaM2C reach higher steady state concentrations than species with Ca^2+^ bound to the N-terminus alone, because Ca^2+^ dissociates from the N-terminus of CaM very rapidly. In addition, CaM2C has a higher affinity for CaMKII than does CaM2N. Once CaM2C binds to the kinase, the affinity of the N-terminus of CaM for Ca^2+^ increases dramatically and, if enough free Ca^2+^ is available, K•CaM4 forms rapidly (Figure 13, red arrows). The three species, K•CaM2C, K•CaM1N2C, and K•CaM4, can all undergo autophosphorylation. However, because the autophosphorylation rate constant of K•CaM4 is 10-fold higher than that of K•CaM2C, the rate of autophosphorylation depends most importantly on 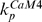 at concentrations of Ca^2+^ above ∼30 µM (Figure 12A and B); whereas, below ∼30 µM Ca^2+^, 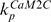 and 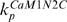 play an equally important role (Figure 12C and D). The prominent role of non-saturated species of CaM under physiological conditions highlights the fact that competition for subsaturating concentrations of Ca^2+^/CaM will often determine the outcome of Ca^2+^ signaling in the spine and likely in many other cell types, as well.

A third significant finding is that when activation of CaMKII is driven by fluctuating Ca^2+^ levels, the dynamics of binding of Ca^2+^ to the K•CaM complex produce a frequency dependence of autophosphorylation. The mechanism of this frequency dependence does not involve the binding of two CaMs to two neighboring subunits in a holoenzyme to produce autophosphorylation, which has been evoked to explain frequency dependence of autophosphorylation of the CaMKII holoenzyme [51]. Rather, it arises from the interaction between the decay constant of the K•CaM2C complex and the interpulse interval of the fluctuating Ca^2+^ stimulus. If the interval is sufficiently short that residual K•CaM2C from one pulse is present at the time of the next pulse, frequency dependence will arise. This mechanism suggests that the contribution of partially filled Ca^2+^/CaM states to activation of autophosphorylation of CaMKII will be more significant when the Ca^2+^ concentration is fluctuating rapidly, as it often does when flowing through ion pores, than it will be during a steady-state jump in Ca^2+^ concentration. It is interesting to note that Ca^2+^/CaM targets could be “tuned” during evolution to respond to varying frequencies of Ca^2+^ stimuli by adjustment of the off rates of Ca^2+^ from the CaM•target complex.

The model presented here will aide the identification and experimental measurement of critical parameters that can be used in constructing more complex models of the CaMKII holoenzyme. It also serves as an example for models of other CaM regulated monomeric enzymes in the spine, and in other cell types, including nitric oxide synthase and calcineurin. Ultimately, the rate constants, optimized with the use of deterministic models like the one presented here, can be translated into probabilities and used for stochastic modeling in a spatially accurate model of a postsynaptic spine with specialized modeling programs such as MCell [55].

## Supporting Information

Text S1 Supplementary Methods
(0.31 MB DOC)

Figure S1 Models of calcium binding to calmodulin. A) Sequential binding model. In this model a state of calmodulin is characterized by the number of calcium ions bound. The dissociation constants are called macroscopic constants. B) Terminal binding model. Here, a state of calmodulin is characterized by the number of calcium ions bound to each of the calmodulin termini. The dissociation constants are called microscopic constants.
(0.62 MB PDF)

Table S1 Parameters for Model S1. Most values are taken from the literature or derived from values in the literature. In a few instances values were derived by fitting to published experimental data as described in Methods.
(0.76 MB PDF)

Table S2 Fitted cooperativity coefficients with their on and off components. The parameters were fit as described in Methods and Figure 4.
(0.04 MB PDF)

Table S3 Sensitivity of autophosphorylation of CaMKII to variations in input parameters. Parameters were varied over a 2.5 fold range from mean of data in Table S1. Sensitivity was measured by partial rank correlation coefficient (PRCC) at different Ca^2+^ concentration ranges. Model parameters are ranked by their PRCC values at 0.1, 1 and 2 seconds after Ca^2+^ addition. Parameters with PRCC values lower than 0.3 are not shown.
(0.68 MB PDF)
