# Supplementary material for: A Dynamic Model of Interactions of Ca2+, Calmodulin, and Catalytic Subunits of Ca2+/Calmodulin-Dependent Protein Kinase II
Source: PLoS Comput Biol. 2010 Feb 12;6(2):e1000675. doi: 10.1371/journal.pcbi.1000675 (PMC2820514; doi:10.1371/journal.pcbi.1000675)
Supplement: Text S1 — Supplementary Methods (0.31 MB DOC) [file pcbi.1000675.s001.doc]

**Text S1**

**Supplemental Methods: Model**

Constraining of kinetic constants for Model 1: Upper layer. The upper layer of the cubic reaction diagram (Figure 2A) represents binding of Ca^2+^ to free CaM, including both association and dissociation reactions. The model defines 8 kinetic rate constants denoted . The four corresponding equilibrium dissociation constants are denoted , such that with and . is the dissociation constant for a calcium ion from the terminus of CaM with ions bound. We refer to these as the “microscopic” dissociation constants to differentiate them from dissociation constants that have been measured without distinguishing which calcium ions bind to each terminus [1-3]. We refer to the latter as “macroscopic” dissociation constants and denote them .

We use three different estimates to obtain ranges for the microscopic dissociation constants. First, we calculated the values of microscopic K*_D_*’s by solving a system of equations relating them to the four experimentally determined macroscopic dissociation constants measured by [4]. The four macroscopic equilibrium dissociation constants (’s) are associated with a model of interaction of Ca^2+^ and CaM that includes five states of Ca^2+^/CaM (Figure S1A). The states are designated by an index corresponding to the number of bound Ca^2+^: CaM0, CaM1, CaM2, CaM3, and CaM4. The microscopic equilibrium dissociation constants (also termed’s) characterize a more refined model that includes nine states of Ca^2+^/CaM: CaM0, CaM1C, CaM1N1C, CaM1N, CaM2C, CaM1N2C, CaM2N, CaM2N1C, CaM4 (see Figure S1B). This model distinguishes between Ca^2+^ ions bound to the N or C termini of CaM but does not distinguish between the two sites at a terminus. It includes the assumption that binding of Ca^2+^ to one terminal is independent of binding of Ca^2+^ to the other terminal. These two assumptions mean that the microscopic state model has only four equilibrium constants as well: . Here we describe how to derive values for the microscopic constants as a function of the macroscopic constants.

For any reaction network, it is possible to write a number (R) of independent equations describing the equilibria between different states, where R is equal to the number of states minus one. (This can be done by excluding just enough reactions from the diagram to remove all loops. One is left with a reaction graph that has no loops or a reaction tree that always has one more state than the number of reactions). Hence, we can write 4 equilibrium relations involving the macroscopic CaM states and 8 relations involving the microscopic CaM states (see below). This yields 12 equations relating the 4 microscopic equilibrium constants to the 4 macroscopic equilibrium constants. There are 12 unknown concentrations of CaM states we would like to eliminate. Eight variables can be eliminated among the 12 equations. Conservation of atoms between the microscopic and macroscopic states (3 equations) plus conservation of mass (one equation) provide the necessary constraints to do so. Explicitly, for given constant [Ca^2+^] and [CaM] one may solve the following equations for the equilibrium dissociation constants:

As a second way of estimating ranges for the microscopic dissociation constants, we use measurements of dissociation constants of Ca^2+^ from mutants engineered to permit Ca^2+^ binding only to the N or C termini [4]. These values are within a factor of two of those we computed for each terminus from the macroscopic values.

As a third way of estimating ranges for the microscopic dissociation constants, we use dissociation constants for Ca^2+^ from individual termini directly measured with the use of tryptic fragments of CaM encompassing the individual N and C termini [3]. These values are also within a factor of two of our computed values. These estimates provide us with narrow ranges of values for the microscopic K_D_’s.

On and off rate constants for the reactions in the upper layer are either taken from direct measurements in the literature or derived from the relationships among measured constants and our calculated microscopic dissociation constants. Off rate constants for dissociation of Ca^2+^ from each CaM terminus with 2 bound Ca^2+^ have been measured experimentally [5,6]. Because of limitations of the temporal resolution of fluorescence measurements in these studies, the reported values were those for the rate limiting step, which is the first dissociation of a Ca^2+^ ion from each terminus (i.e. ). We use these constants, and our calculated microscopic and , to calculate and . To calculate on and off rate constants for binding of the first Ca^2+^ to each terminus, we assume that the observed cooperativity between binding of Ca^2+^ to the first and second sites results entirely from a change in off rates. This assumption effectively defines and . We then estimate the corresponding off rates by multiplying the on rates by the calculated microscopic affinity constants and . The resulting 8 kinetic rate constants, which are required to specify binding of Ca^2+^ to free CaM in the upper layer of Model 1 (Text Figure 2A and Figure S1), are listed in Table S1.

Example of a calculation of equilibrium constants based on the principle of microscopic reversibility: Consider the reaction loop specifying reactions among CaM4, CaM1N2C, K•CaM1N2C, and K•CaM4 illustrated in Text Figure 3. This loop defines the thermodynamic relationships that we use to estimate affinities of free Ca^2+^ for K•CaM1N2C and of CaM1N2C for CaMKII. The dissociation constants for the four reactions in the loop are first expressed in terms of free energies,

Because around the loop (microscopic reversibility), , yielding a relationship that we use to calculate .

Fitting of *s* and *r* for on and off rate cosntants: We estimated the on rate constants (, , , ) and ( ) as well as the corresponding off rate constants by generating ODEs representing the relations in Model 1. For these calculations, we represent *s* and *r* as having contributions from both the on and the off rate constants. For example, the equations represent the dissociation of a single Ca^2+^ from the N terminus, and represent the dissociation of the first Ca^2+^ from the N terminus when two Ca^2+^ ions are bound. Analogous equations represent dissociation of the two Ca^2+^ ions from the C terminus. Recall that we showed in the main text that the cooperativity coefficients (*s* and *r*) for binding of Ca^2+^ to K•CaM*n*N*c*C are equal to those for binding of CaM*n*N*c*C to K (which we designate and for clarity in the following derivations). Because and (and thus, and ) are determined from experimental measurements, we are left with 4 additional pairs of parameters to estimate, constrained by the four relations , , and ). The simplex method for gradient descent was used to fit the parameters to three sets of experimental data. Specifically, we fit experimental data for dissociation of CaM from Ca^2+^•CaM•CaMKII in high (Text Figure 3A) and in low (Text Figure 3B) Ca^2+^ [data from Figure 2B in 7] and for dissociation of Ca^2+^ from Ca^2+^•CaM•CaMKII [Text Figure 3C, data from Fig. 4A in 8]. Fits were made under three different sets of constraints: constant on rates () and varying off rates (Text Figure 3, green), constant off rates () and varying on rates (Text Figure 3, blue), and varying both on and off rates simultaneously (Text Figure 3, red). The objective function was taken as the sum of the squares of differences between simulations and experiments for each of the three experiments. The best fit was obtained when the on and off components of the cooperativity coefficients were allowed to vary simultaneously (Table S2 and Text Figure 3, red). The constants providing the best fit converged to nearly identical values when we began the regression at several different starting points, suggesting that the equilibrium relationships are satisfied by a single narrow range of parameter values.

Model 1 (Kennedy2010_Ca2+_CaM_mCaMKII) is available in SBML format in the Biomodels Database located at [www.biomodels.net](http://www.biomodels.net/) [9].

Derivation of parameters for Model 2: Coarse grained model. Binding of two Ca^2+^ ions to each of the two termini of CaM is highly cooperative; binding of one Ca^2+^ to either terminus enhances the affinity of that terminus for a second Ca^2+^. We have created a model that treats the binding of a pair of Ca^2+^ ions to a terminus as a single event. We derive the equilibrium binding constants and rate constants from those in Model 1 using a steady state approximation applied to the concentrations of CaM species with odd numbers of bound Ca^2+^. For example, the following reactions describe interaction of Ca^2+^ with the C terminus of CaM in Model 1:

When Ca^2+^ binds with high cooperativity, the equilibrium for the second reaction is far to the right and the concentration of the intermediate CaM*n*N1C is close to zero. Thus, we can approximate the reaction in Model 2 as:

,

where the subscript *ss* (for steady-state) indicates that the rate constant is derived by expressing the relations in Model 1 as differential equations and setting d[CaM*n*N1C]/dt = 0 as dictated by the steady state approximation. Making an analogous approximation for the interaction of Ca^2+^ with the N terminus and for binding of Ca^2+^ to K•CaM*n*N*c*C yields the model for interactions among Ca^2+^, CaM, and CaMKII shown in Text Figure 5. The derived expressions for the on and off rates for interactions of pairs of Ca^2+^ with the C-terminus of CaM are:

The same reasoning yields similar expressions for the on and off rates for interactions of Ca^2+^ with the N terminus and for interactions among Ca^2+^, CaM, and CaMKII.

Consider the sequential Ca^2+^ binding reactions to the CaM C terminal:

We use the Law of Mass Action to write differential equations governing the time evolution of the CaM species concentrations, *i.e.*

At sufficiently high concentrations of Ca^2+^ compared to CaM*,* . We will assume this to be the case, consistent with a model in which pairs of Ca^2+^ bind to the termini nearly simultaneously (such binding becomes increasingly unlikely at low concentrations). Further, if binding of two Ca^2+^ ions at the C terminal is cooperative, then the concentration of the intermediate species with one Ca^2+^ bound, [CaM1C] will be very low. Thus we assume . The latter embodies the steady state approximation for the system. Setting the right hand side of Equation 0.2 equal to zero yields the following expression for [CaM1C]:

Substituting this equation into equations (0.1) and (0.3) and rearranging terms leads to:

Interpreting these equations as the Mass Action derivative expressions for the reaction

,

where the subscript *ss* refers to the steady state assumption, one can deduce

The rates for binding of Ca^2+^ to the N terminal of CaM can be derived in a similar fashion. Substituting the appropriate parameters from Model 1 for leads to expressions for the kinetic rates in Model 2 as a function of the rates in Model 1.

Constraining of parameters for phosphorylation reactions: Autophosphorylation of monomeric CaMKII (mCaMKII) occurs via an intersubunit reaction. Two molecules of K•CaM*n*N*c*C bind to each other, after which one acts as catalyst and the other acts as substrate [Text Figure 6, 10]. We obtain afor formation of a complex of two K•CaM4 species by fitting autophosphorylation data from [Figure 5 in 11, supplementary Figure S2]. Attempts to constrain values for and by fitting this data revealed that the fits depend only on the and are not sensitive to the values of or . Therefore, we assume a commonly used value for the on rate constant for diffusion-limited protein-protein interactions, = 50 µM^-1^s^-1^ [see 12]. Because there is no data measuring dissociation constants for pairs of K•CaM*n*N*c*C for (*n*, *c* <4), we assume that all pairs have the same dissociation constant.

A K•CaM*n*N*c*C molecule in a monomer-monomer complex is autophosphorylated with a rate constant dependent on the number of Ca^2+^ ions bound to the CaM associated with the monomer. For K•CaM4, the autophosphorylation rate constant () is set equal to the autophosphorylation rate measured when the CaMKII holoenzyme is fully saturated with CaM4 and ATP [4]. Autophosphorylation rates have also been measured when the CaMKII holoenzyme was saturated with two mutant CaMs [CaM2N-WT and CaM2C-WT, 4] that mimic K•CaM2N and K•CaM2C, respectively. We set the autophosphorylation rates of K•CaM2N and K•CaM2C ( and ) equal to these corresponding rates. The autophosphorylation rates for K•CaM*n*N*c*C with odd numbers of bound Ca^2+^ are assigned a range of values between those that have been estimated experimentally. For example, the value of has a range between zero and the higher value of as seen in Table S1; the value of has a range between and .

Latin Hypercube Sampling: For our implementation of Latin Hypercube sampling, we assumed a uniform distribution of the values of input parameters over the specified range. The method divides values for each parameter into N equally probable bins. A single random value from each bin is chosen and is placed in a vector of N values for that parameter (typically N=1000) after which the order of the values is randomized. The vectors for each parameter are then assembled into an N by M matrix, where M is the number of parameters to be tested. Thus, each of the N columns of the matrix contains a randomized set of values for an individual parameter.

The concentration ranges of mCaMKII and CaM that we used in the sensitivity analysis are meant to bracket estimated concentrations within spines *in vivo* (see Methods). They are considerably higher than commonly used in experiments; thus, Ca^2+^ cannot saturate binding to CaM until it reaches 160 µM (4 X 40µM, the highest CaM concentration).

Calculation of PRCC: We calculated the PRCC between each input parameter and autophosphorylation of CaMKII (our output of interest) over the time of the simulation. First, the sum of the concentrations of all autophosphorylated CaMKII species was calculated for the sets of values in each row of the N x M matrix. These values were added to the matrix to generate a new N by M+1 matrix. A rank matrix was generated by assigning the values in each column a rank value based on their magnitude. A correlation matrix was then generated from the rank matrix wherein the correlation between all parameters was calculated according to the Spearman’s rank correlation coefficient. PRCC values for all parameters were calculated from the correlation matrix by matrix inversion. The 95% confidence intervals of PRCC values for each parameter were calculated according to methods described in [13].

References

1. Crouch TH, Klee CB (1980) Positive cooperative binding of calcium to bovine brain calmodulin. Biochemistry 19: 3692-3698.

2. Klee CB (1988) Interaction of calmodulin with Ca^2+^ and target proteins. In: Cohen P, Klee CB, editors. Calmodulin. Amsterdam: Elsevier. pp. 35-56.

3. Linse S, Helmersson A, Forsen S (1991) Calcium binding to calmodulin and its globular domains. J Biol Chem 266: 8050-8054.

4. Shifman JM, Choi MH, Mihalas S, Mayo SL, Kennedy MB (2006) Ca^2+^/Calmodulin-dependent protein kinase II (CaMKII) is activated by calmodulin with two bound calciums. Proc Natl Acad Sci USA 103: 13968-13973.

5. Peersen OB, Madsen TS, Falke JJ (1997) Intermolecular tuning of calmodulin by target peptides and proteins: differential effects on Ca(2+) binding and implications for kinase activation. Protein Science 6: 794-807.

6. Brown SE, Martin SR, Bayley PM (1997) Kinetic control of the dissociation pathway of calmodulin-peptide complexes. J Biol Chem 272: 3389-3397.

7. Meyer T, Hanson PI, Stryer L, Schulman H (1992) Calmodulin trapping by calcium-calmodulin-dependent protein kinase. Science 256: 1199-1202.

8. Gaertner TR, Putkey JA, Waxham MN (2004) RC3/Neurogranin and Ca^2+^/calmodulin-dependent protein kinase II produce opposing effects on the affinity of calmodulin for calcium. J Biol Chem 279: 39374-39382.

9. Le Novere N, Bornstein B, Broicher A, Courtot M, Donizelli M, et al. (2006) BioModels Database: A free centralized database of curated, published, quantitative kinetic models of biochemical and cellular systems. Nucleic Acids Res 34: D689-D691.

10. Hanson PI, Meyer T, Stryer L, Schulman H (1994) Dual role of calmodulin in autophosphorylation of multifunctional CaM kinase may underlie decoding of calcium signals. Neuron 12: 943-956.

11. Bradshaw JM, Hudmon A, Shulman H (2002) Chemical Quenched Flow Kinetic Studies Indicate an Intraholoenzyme Autophosphorylation Mechanism for Ca^2+^. J Biol Chem 277: 20991-20998.

12. Copeland RA (2000) Enzymes. New York: Wiley.

13. Howell DC (1987) Statistical Methods for Psychology. Boston: Duxbury Press. p242 p.
