# Supplementary figures and images for: A Dynamic Model of Interactions of Ca2+, Calmodulin, and Catalytic Subunits of Ca2+/Calmodulin-Dependent Protein Kinase II
Source: PLoS Comput Biol. 2010 Feb 12;6(2):e1000675. doi: 10.1371/journal.pcbi.1000675 (PMC2820514; doi:10.1371/journal.pcbi.1000675)

**A**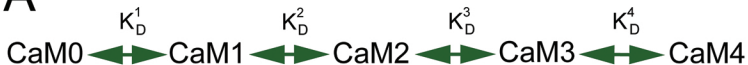**B**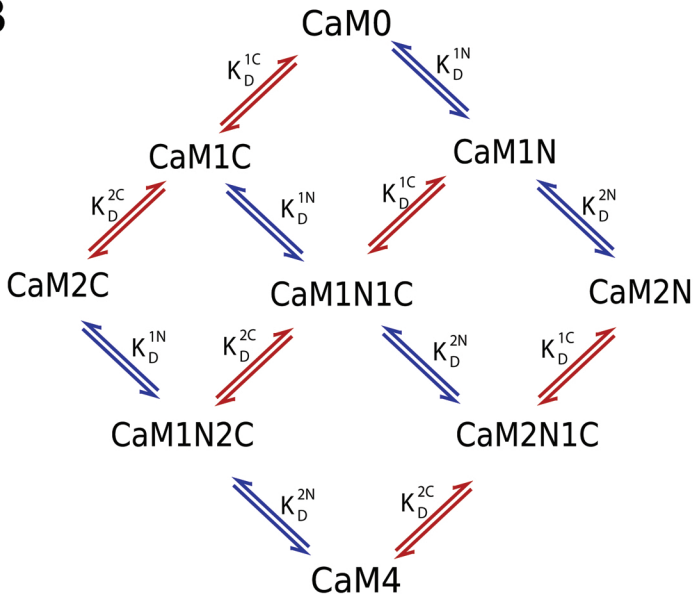

Supplement: Figure S1 — Models of calcium binding to calmodulin. A) Sequential binding model. In this model a state of calmodulin is characterized by the number of calcium ions bound. The dissociation constants are called macroscopic constants. B) Terminal binding model. Here, a state of calmodulin is characterized by the number of calcium ions bound to each of the calmodulin termini. The dissociation constants are called microscopic constants. (0.62 MB PDF) [file pcbi.1000675.s002.pdf]
