# Supplementary material for: A Dynamic Model of Interactions of Ca2+, Calmodulin, and Catalytic Subunits of Ca2+/Calmodulin-Dependent Protein Kinase II
Source: PLoS Comput Biol. 2010 Feb 12;6(2):e1000675. doi: 10.1371/journal.pcbi.1000675 (PMC2820514; doi:10.1371/journal.pcbi.1000675)
Supplement: Table S1 — Parameters for Model S1. Most values are taken from the literature or derived from values in the literature. In a few instances values were derived by fitting to published experimental data as described in Methods. (0.76 MB PDF) [file pcbi.1000675.s003.pdf]

Table S1. Parameter Values for Model 1

Most values are taken from the literature or derived from values in the literature. In a few instances values were derived by fitting to published experimental data as described in Methods.

Parameter	Description	Value Range	Reference
**Ca^{2+} binding to CaM**			
K_D^{1C}	Equilibrium binding of 1st Ca^{2+} to CaM C-terminus	7.3 - 12 uM	[1-3]
k_{off}^{1C}	Ca^{2+} dissociation from CaM C-terminus	10 - 70 s^{-1}	[4-6]
k_{on}^{1C}	Ca^{2+} binding to CaM C-terminus	1.2 - 9.6 $uM^{-1}s^{-1}$	[7]
K_D^{2C}	Equilibrium binding of 2nd Ca^{2+} to CaM C-terminus	0.4 - 1.7 uM	[1-3]
k_{off}^{2C}	2nd Ca^{2+} dissociation from CaM C-terminus	8.5 - 10 s^{-1}	[4]
k_{on}^{2C}	2nd Ca^{2+} binding to CaM C-terminus	5 - 25 $uM^{-1}s^{-1}$	[7]
K_D^{1N}	Equilibrium binding of 1st Ca^{2+} to CaM N-terminus	15 - 40 uM	[1-3]
k_{off}^{1N}	1st Ca^{2+} dissociation from CaM N-terminus	1000 - 4000 s^{-1}	[4,6,8,9]
k_{on}^{1N}	1st Ca^{2+} binding to CaM N-terminus	25 - 260 $uM^{-1}s^{-1}$	[7]
K_D^{2N}	Equilibrium binding of 2nd Ca^{2+} to CaM N-terminus	3.3 - 9 uM	[1-3]
k_{off}^{2N}	2nd Ca^{2+} dissociation from CaM N-terminus	500 - >1000 s^{-1}	[4-6,8,9]
k_{on}^{2N}	2nd Ca^{2+} binding to CaM N-terminus	50 - 300 $uM^{-1}s^{-1}$	[7]
**CaM binding to CaMKII**			
k_{on}^{CaM0}	CaM0 binding to CaMKII	3.8 $mM^{-1}s^{-1}$	[10]
k_{off}^{CaM0}	CaM0 dissociating from CaMKII	5.5 s^{-1}	[10]
k_{on}^{CaM1C}	CaM1C binding to CaMKII	59 $mM^{-1}s^{-1}$	[10]
k_{off}^{CaM1C}	CaM1C dissociating from CaMKII	6.1 s^{-1}	[10]
K_D^{CaM2C}	Equilibrium binding of CaM2C to CaMKII	1.6 - 8.4 uM	[2]
k_{on}^{CaM2C}	CaM2C binding to CaMKII	0.92 $uM^{-1}s^{-1}$	[10]
k_{off}^{CaM2C}	CaM2C dissociating from CaMKII	6.8 s^{-1}	[10]
$k_{on}^{CaM1N1C}$	CaM1N1C binding to CaMKII	0.33 $uM^{-1}s^{-1}$	[10]
$k_{off}^{CaM1N1C}$	CaM1N1C dissociating from CaMKII	3.4 s^{-1}	[10]
$k_{on}^{CaM1N2C}$	CaM1N2C binding to CaMKII	5.2 $uM^{-1}s^{-1}$	[10]
$k_{off}^{CaM1N2C}$	CaM1N2C dissociating from CaMKII	3.8 s^{-1}	[10]
k_{on}^{CaM1N}	CaM1N binding to CaMKII	22 $mM^{-1}s^{-1}$	[10]
k_{off}^{CaM1N}	CaM1N dissociating from CaMKII	3.1 s^{-1}	[10]
K_D^{CaM2N}	Equilibrium binding of CaM2N to CaMKII	16.5 - 23.5 uM	[2]
k_{on}^{CaM2N}	CaM2N binding to CaMKII	0.12 $uM^{-1}s^{-1}$	[10]
k_{off}^{CaM2N}	CaM2N dissociating from CaMKII	1.7 s^{-1}	[10]

$k_{on}^{CaM\,2N1C}$	CaM2N1C binding to CaMKII	1.9 $uM^{-1}s^{-1}$	[10]
$k_{off}^{CaM\,2N1C}$	CaM2N1C dissociating from CaMKII	1.9 s^{-1}	[10]
$K_{D}^{CaM\,4}$	Equilibrium binding of CaM4 to CaMKII	0.04 - 0.08 uM	[2,4,11-13]
$k_{on}^{CaM\,4}$	CaM4 binding to CaMKII	14 - 60 $uM^{-1}s^{-1}$	[7]
$k_{off}^{CaM\,4}$	CaM4 dissociating from CaMKII	1.1 - 2.3 s^{-1}	[14]
**Ca^{2+} binding to CaM-CaMKII**			
k_{off}^{K1C}	1st Ca^{2+} dissociation from K•CaM C-terminus	33 s^{-1}	[15]
k_{on}^{K1C}	1st Ca^{2+} binding to K•CaM C-terminus	44 $uM^{-1}s^{-1}$	[15]
k_{off}^{K2C}	2nd Ca^{2+} dissociation from K•CaM C-terminus	0.49 - 4.9 s^{-1}	[4,8,14,15]
k_{on}^{K2C}	2nd Ca^{2+} binding to K•CaM C-terminus	44 $uM^{-1}s^{-1}$	[15]
k_{off}^{K1N}	1st Ca^{2+} dissociation from K•CaM N-terminus	300 s^{-1}	[15]
k_{on}^{K1N}	1st Ca^{2+} binding to K•CaM N-terminus	76 $uM^{-1}s^{-1}$	[15]
k_{off}^{K2N}	2nd Ca^{2+} dissociation from K•CaM N-terminus	6 - 60 s^{-1}	[4,8,14,15]
k_{on}^{K2N}	2nd Ca^{2+} binding to K•CaM N-terminus	76 $uM^{-1}s^{-1}$	[15]
**CaMKII autophosphorylation**			
K_{D}^{CaMKII}	Equilibrium binding of two CaMKII subunits	1.3 - 45 uM	[16,17]
k_{on}^{CaMKII}	K•CaM binding to KCaM	10 - 100 $uM^{-1}s^{-1}$	[18,19]
k_{off}^{CaMKII}	K•CaM dissociation from KCaM	13 - 4500 s^{-1}	[7]
$k_{on}^{CaMKIIp}$	K•CaM binding to pKCaM	10 - 100 $uM^{-1}s^{-1}$	[18,19]
$k_{off}^{CaMKIIp}$	K•CaM dissociation from pKCaM	13 - 4500 s^{-1}	[7]
$k_{p}^{CaM\,0}$	Autophosphorylation of KCaM0	0 s^{-1}	[20]
$k_{p}^{CaM\,1C}$	Autophosphorylation of KCaM1C	0.0 - 0.084 s^{-1}	[20]
$k_{p}^{CaM\,2C}$	Autophosphorylation of KCaM2C	0.044 - 0.084 s^{-1}	[2]
$k_{p}^{CaM\,1N1C}$	Autophosphorylation of KCaM1N1C	0.0 - 0.16 s^{-1}	[20]
$k_{p}^{CaM\,1N2C}$	Autophosphorylation of KCaM1N2C	0.044 - 1.25 s^{-1}	[20]
$k_{p}^{CaM\,1N}$	Autophosphorylation of KCaM1N	0.0 - 0.16 s^{-1}	[20]
$k_{p}^{CaM\,2N}$	Autophosphorylation of KCaM2N	0.079 - 0.16 s^{-1}	[2]
$k_{p}^{CaM\,2N1C}$	Autophosphorylation of KCaM2N1C	0.079 - 1.25 s^{-1}	[20]
$k_{p}^{CaM\,4}$	Autophosphorylation of KCaM4	0.5 - 1.25 s^{-1}	[2,21]

1. Linse S, Helmersson A, Forsen S (1991) Calcium binding to calmodulin and its globular domains. J Biol Chem 266: 8050-8054.
2. Shifman JM, Choi MH, Mihalas S, Mayo SL, Kennedy MB (2006) Ca^{2+}/Calmodulin-dependent protein kinase II (CaMKII) is activated by calmodulin with two bound calciums. Proc Natl Acad Sci U S A 103: 13968-13973.

3. Kubota Y, Putkey J, Waxham M (2007) Neurogranin controls the spatiotemporal pattern of postsynaptic Ca^{2+}/CaM signaling. Biophys J 93: 3848-3859.
4. Gaertner TR, Putkey JA, Waxham MN (2004) RC3/Neurogranin and Ca^{2+}/calmodulin-dependent protein kinase II produce opposing effects on the affinity of calmodulin for calcium. J Biol Chem 279: 39374-39382.
5. Martin S, Maune J, Beckingham K, Bayley P (1992) Stopped-flow studies of calcium dissociation from calcium-binding-site mutants of Drosophila melanogaster calmodulin. European Journal of Biochemistry 205: 1107-1114.
6. Persechini A, White HD, Gansz KJ (1996) Different mechanisms for Ca^{2+} dissociation from complexes of calmodulin with nitric oxide synthase or myosin light chain kinase. J Biol Chem 271: 62-67.
7. Calculated from the relationship $K_D = k_{off}/k_{on}$.
8. Peersen OB, Madsen TS, Falke JJ (1997) Intermolecular tuning of calmodulin by target peptides and proteins: differential effects on Ca^{2+} binding and implications for kinase activation. Protein Sci 6: 794-807.
9. Brown SE, Martin SR, Bayley PM (1997) Kinetic control of the dissociation pathway of calmodulin-peptide complexes. J Biol Chem 272: 3389-3397.
10. Determined in this work. Fit to experimental time series data in reference 13.
11. Rosenberg OS, Deindl S, Sung R-J, Nairn AC, Kuriyan J (2005) Structure of the autoinhibited kinase domain of CaMKII and SAXS analysis of the holoenzyme. 0092-8674 123: 849-860.
12. De Koninck P, Schulman H (1998) Sensitivity of CaM Kinase II to the frequency of Ca^{2+} oscillations. Science 279: 227-230.
13. Meyer T, Hanson PI, Stryer L, Schulman H (1992) Calmodulin trapping by calcium-calmodulin-dependent protein kinase. Science 256: 1199-1202.
14. Singla SI, Hudmon A, Goldberg JM, Smith JL, Schulman H (2001) Molecular characterization of calmodulin trapping by calcium/calmodulin-dependent protein kinase II. J Biol Chem 276: 29353-29360.
15. Determined in this work. Fit to experimental time series data in reference 4.
16. Hanson PI, Meyer T, Stryer L, Schulman H (1994) Dual role of calmodulin in autophosphorylation of multifunctional CaM kinase may underlie decoding of calcium signals. Neuron 12.
17. Determined in this work. Fit to experimental time series data in Bradshaw JM, Hudmon A, Shulman H (2002) Chemical Quenched Flow Kinetic Studies Indicate an Intraholoenzyme Autophosphorylation Mechanism for Ca^{2+}. J Biol Chem 277: 20991-20998.
18. Assumed to be slightly less than an ideal (diffusion only limited) interaction between enzyme and substrate; as reported in Copeland, Enzymes, 2000.
19. Copeland R (2000) Enzymes: a practical introduction to structure, mechanism, and data analysis. New York: Wiley-VCH. 121 p.
20. Extrapolated from published values in reference 2.
21. Miller SG, Kennedy MB (1986) Regulation of brain Type II Ca^{2+}/Calmodulin-dependent protein kinase by autophosphorylation: a Ca^{2+}-triggered molecular switch. 0092-8674 44: 861-870.
