# Supplementary material for: A Dynamic Model of Interactions of Ca2+, Calmodulin, and Catalytic Subunits of Ca2+/Calmodulin-Dependent Protein Kinase II
Source: PLoS Comput Biol. 2010 Feb 12;6(2):e1000675. doi: 10.1371/journal.pcbi.1000675 (PMC2820514; doi:10.1371/journal.pcbi.1000675)
Supplement: Table S2 — Fitted cooperativity coefficients with their on and off components. The parameters were fit as described in Methods and Figure 4. (0.04 MB PDF) [file pcbi.1000675.s004.pdf]

Table S2. Fitted cooperativity coefficients with their on and off components

The parameters were fit as described in Methods and in Text Figure 4.

s	10
r	14
s_{on}	0.8
s_{off}	12.5
r_{on}	8.75
r_{off}	1.6
$s_{d,on}$	5.6
$s_{d,off}$	1.8
$r_{d,on}$	8.75
$r_{d,off}$	0.9
