# Supplementary material for: A Dynamic Model of Interactions of Ca2+, Calmodulin, and Catalytic Subunits of Ca2+/Calmodulin-Dependent Protein Kinase II
Source: PLoS Comput Biol. 2010 Feb 12;6(2):e1000675. doi: 10.1371/journal.pcbi.1000675 (PMC2820514; doi:10.1371/journal.pcbi.1000675)
Supplement: Table S3 — Sensitivity of autophosphorylation of CaMKII to variations in input parameters. Parameters were varied over a 2.5 fold range from mean of data in Table S1. Sensitivity was measured by partial rank correlation coefficient (PRCC) at different Ca2+ concentration ranges. Model parameters are ranked by their PRCC values at 0.1, 1 and 2 seconds after Ca2+ addition. Parameters with PRCC values lower than 0.3 are not shown. (0.68 MB PDF) [file pcbi.1000675.s005.pdf]

Table S3. Sensitivity of autophosphorylation of CaMKII to variations in input parameters (varied over a 2.5 fold range from mean of data in Table S1) as measured by partial rank correlation coefficient (PRCC) at different Ca^{2+} concentration ranges. Model parameters are ranked by their PRCC values at 0.1, 1 and 2 seconds after Ca^{2+} addition. Parameters with PRCC values lower than 0.3 are not shown.

**0.1 sec**			**1 sec**			**2 sec**		
**Parameter**	**Confidence Interval**		**Parameter**	**Confidence Interval**		**Parameter**	**Confidence Interval**	
**Low Ca^{2+} (1 - 5 µM)**								
$[Ca^{2+}]$	0.78	0.73	$[Ca^{2+}]$	0.78	0.73	$[Ca^{2+}]$	0.78	0.73
k_p^{CaM1C}	0.73	0.66	k_p^{CaM1C}	0.72	0.66	k_p^{CaM1C}	0.72	0.65
k_p^{CaM1N}	0.64	0.56	[CaM]	0.64	0.56	[CaM]	0.64	0.57
[CaM]	0.63	0.55	k_p^{CaM2C}	0.57	0.48	k_p^{CaM2C}	0.60	0.52
k_{on}^{K1N}	0.43	0.32	k_p^{CaM1N}	0.48	0.38	k_p^{CaM1N}	0.46	0.36
k_p^{CaM2N}	0.40	0.29	k_{on}^{K1N}	0.46	0.36	k_{on}^{K1N}	0.45	0.35
k_{on}^{K1C}	-0.29	-0.40	k_{off}^{K1N}	-0.36	-0.46	k_{off}^{K1N}	-0.35	-0.45
k_{off}^{K1N}	-0.39	-0.49	K_D	-0.62	-0.69	K_D	-0.62	-0.69
K_D	-0.64	-0.71						
**Medium Ca^{2+} (10 - 50 µM)**								
$[Ca^{2+}]$	0.78	0.73	$[Ca^{2+}]$	0.78	0.73	$[Ca^{2+}]$	0.78	0.73
k_p^{CaM4}	0.65	0.58	k_p^{CaM2C}	0.59	0.51	k_p^{CaM2C}	0.63	0.55
$k_p^{CaM2N1C}$	0.61	0.53	k_{on}^{K1N}	0.53	0.43	k_{on}^{K1N}	0.53	0.43
$k_p^{CaM1N2C}$	0.38	0.27	$k_p^{CaM1N2C}$	0.46	0.36	$k_p^{CaM1N2C}$	0.48	0.37
k_p^{CaM2N}	0.37	0.26	k_p^{CaM4}	0.46	0.36	k_{off}^{K1C}	0.43	0.32
k_p^{CaM1C}	0.36	0.25	k_{off}^{K1C}	0.43	0.32	k_{off}^{K2C}	0.41	0.30
k_{on}^{K2N}	0.36	0.25	k_p^{CaM1C}	0.39	0.28	k_p^{CaM1C}	0.40	0.30
k_{off}^{K2N}	-0.26	-0.37	k_{on}^{K2N}	0.38	0.27	k_p^{CaM4}	0.40	0.29
K_D	-0.63	-0.70	k_{off}^{K2N}	-0.28	-0.39	k_{on}^{K2C}	-0.31	-0.42
			k_{on}^{K2C}	-0.31	-0.42	k_{on}^{K1C}	-0.32	-0.42
			k_{on}^{K1C}	-0.34	-0.44	k_{off}^{K1N}	-0.43	-0.52
			k_{off}^{K1N}	-0.42	-0.52	K_D	-0.61	-0.68
			K_D	-0.61	-0.69			
**High Ca^{2+} (50 - 250 µM)**								
$[Ca^{2+}]$	0.68	0.61	$[Ca^{2+}]$	0.68	0.60	$[Ca^{2+}]$	0.68	0.60
k_p^{CaM4}	0.64	0.56	k_p^{CaM4}	0.58	0.50	[CaM]	0.55	0.46
[CaM]	0.54	0.44	[CaM]	0.52	0.43	k_p^{CaM4}	0.54	0.45
